# Functional organization outweighs spatial separation in structuring microbial communities across a petroleum-hydrocarbon groundwater plume

**DOI:** 10.3389/fmicb.2026.1938677

**Published:** 2026-09-16

**Authors:** Ruihuan Zhang, Yu Chen, Deyi Hou

**Affiliations:** 1 School of Environment, Tsinghua University, Beijing, China; 2 State Key Laboratory of Regional Environment and Sustainability, Tsinghua University, Beijing, China

**Keywords:** functional diversity, groundwater, hydrogeology, metagenomics, microbial community, petroleum hydrocarbons

## Abstract

**Objective:**

Petroleum-hydrocarbon plumes in groundwater are shaped by hydrological transport, redox conditions, and indigenous microbial activity. However, it remains unclear whether spatial differences among groundwater microbiomes primarily reflect physical separation among wells or ecological reorganization associated with distinct attenuation states within a plume.

**Methods:**

We integrated hydrogeological context, petroleum-hydrocarbon concentrations, hydrochemical variables, and shotgun-metagenomic profiles from 15 groundwater monitoring wells at a petroleum-hydrocarbon-impacted site. Community beta diversity, spatial and functional distance relationships, permutation-based group tests, and exploratory composite indices of natural attenuation potential and taxonomic-functional diversity were evaluated.

**Results:**

Genus-level community dissimilarity increased with estimated spatial distance (Mantel r = 0.240, *p* = 0.034), but was more strongly associated with functional-module dissimilarity (r = 0.670, *p* = 0.001). Biodegradation potential remained associated with community dissimilarity after controlling for spatial distance (partial Mantel *r* = 0.428, *p* = 0.001). Operational attenuation states explained 35.4% of genus-level compositional variation (PERMANOVA *p* = 0.008). High natural attenuation potential and high taxonomic-functional diversity occurred in partly distinct wells.

**Conclusion:**

The results indicate that groundwater microbiome turnover within the plume is spatially structured but is more closely coupled to functional reorganization than to distance alone. Joint consideration of hydrological setting, microbial degradation potential, and functional diversity may improve groundwater natural-attenuation assessment.

## 1 Introduction

Petroleum hydrocarbon releases remain a persistent threat to groundwater quality because dissolved aromatic hydrocarbons and residual petroleum fractions can migrate through aquifers while undergoing dilution, sorption, phase partitioning, and biodegradation (Cozzarelli et al., 2022; Thornton et al., 2024). Their environmental fate is therefore governed not only by contaminant loading, but also by groundwater flow, aquifer structure, redox conditions, and microbial metabolism (Foster and Chilton, 2003; Chapelle, 2000). Monitored natural attenuation is consequently an important management option for petroleum-impacted groundwater (Song et al., 2023; Thornton et al., 2024). However, a defensible evaluation requires evidence that concentration changes reflect attenuation processes rather than dilution or mixing alone (Chiu et al., 2013; Bruckberger et al., 2020).

Microbial transformation is a central component of petroleum-hydrocarbon attenuation in aquifers. Hydrocarbon inputs can alter electron-acceptor availability and generate spatially structured redox regimes, leading to distinct assemblages of aerobic degraders, nitrate reducers, iron reducers, sulfate reducers, fermenters, and methanogens. Field studies of crude-oil and BTEX plumes have demonstrated that microbial populations, catabolic genes, and redox processes can vary substantially across contaminant source zones, plume margins, and downgradient groundwater (Bekins et al., 2001; Hendrickx et al., 2005; Winderl et al., 2008). The variation is ecologically meaningful, but its interpretation remains difficult. For example, differences in petroleum hydrocarbon concentrations among wells may arise from biodegradation, whereas at other sites they are dominated by hydrological mixing or dilution with less contaminated groundwater (Chiu et al., 2013; Bruckberger et al., 2020). Concentration data alone are therefore insufficient to resolve whether a well represents high contaminant pressure, an actively responding microbial community, or a residual state with legacy functional capacity.

Another challenge concerns the spatial organization of groundwater microbiomes. Groundwater microbial communities are influenced by aquifer lithology, flow regime, recharge, physicochemical selection, and dispersal limitation (Griebler and Lueders, 2009; Lehman et al., 2001). Studies of pristine and contaminated aquifers have shown that hydrochemistry and spatial separation can jointly structure community composition, although their relative contributions differ among hydrogeological settings (Yan et al., 2020; Merino et al., 2022; Groult et al., 2022). Environmental stress can further alter the balance between stochastic and deterministic assembly processes in groundwater (Ning et al., 2024). These findings indicate that it remains insufficiently resolved whether differences among groundwater microbial communities in petroleum-hydrocarbon plumes primarily reflect physical separation and hydrological connectivity or ecological reorganization associated with hydrocarbon transformation and attenuation state. Recent work has emphasized the value of integrating groundwater biogeochemistry with microbial information when characterizing subsurface connectivity, but this integration is still rarely developed as a function-oriented attenuation framework (Acciardo et al., 2025).

Shotgun metagenomics provides an opportunity to address this gap because it can characterize taxonomic composition and encoded functional potential simultaneously. Metagenomic studies have revealed substantial shifts in community structure, metabolic potential, and resistance or degradation-related genes between pristine and contaminated groundwater systems (Hemme et al., 2010; Hemme et al., 2015). In petroleum-impacted aquifers, metagenomic analyses have identified diverse microbial assemblages and hydrocarbon-degradation capacities, while genome-resolved studies of engineered groundwater-treatment systems have further shown that community composition and aromatic-degradation pathways can differ strongly across redox and treatment conditions (Kachienga et al., 2018; Hauptfeld et al., 2022). Microbial and functional biomarkers can therefore complement conventional geochemical measurements for contamination assessment (Fields et al., 2006; Bagwell et al., 2025). However, it should be noted that the detection of a gene or pathway indicates metabolic potential rather than *in situ* activity.

Beyond differences in microbial community composition, two complementary dimensions are relevant for evaluating attenuation across a contaminated groundwater plume. The first is natural attenuation potential, which in this study refers to the relative strength of a site-specific attenuation response and is operationally represented by the natural attenuation potential index (NAPI). NAPI integrates contaminant pressure, biological response, degradation-associated taxonomic and functional abundance, and redox-resource context, and is therefore intended as a comparative indicator rather than a direct estimate of *in situ* degradation rate. The second dimension is degradation-associated taxonomic-functional diversity, which describes the richness and diversity of hydrocarbon-degradation-related taxa, pathways, and genes. Unlike functional redundancy in its strict ecological sense (Louca et al., 2018), taxonomic-functional diversity does not assume functional interchangeability among taxa, but instead characterizes how broadly degradation-related taxonomic and functional features are represented within the community. These two dimensions capture different ecological properties. NAPI emphasizes the strength of the contemporary pressure-response relationship, whereas taxonomic-functional diversity reflects the breadth and diversity of the encoded degradation-associated repertoire.

Here, we integrated hydrogeological context, petroleum-hydrocarbon content, physicochemical variables, and shotgun-metagenomic profiles from 15 monitoring wells in a petroleum-hydrocarbon-impacted groundwater system. We first evaluated whether genus-level microbial community turnover was more strongly associated with spatial separation or with functional organization and biodegradation potential. We then used an operational attenuation-state framework to summarize joint variation in pollutant pressure, biological response, and metagenome-derived functional information across the monitoring network. Within this framework, we compared natural attenuation potential with degradation-associated taxonomic-functional diversity to determine whether strong contemporary attenuation responses coincided spatially with broad degradation-associated taxonomic and functional repertoires. By integrating these spatial, taxonomic, and functional dimensions, the study provides a site-scale perspective on how microbial community organization and degradation-related functional potential vary across a heterogeneous petroleum-hydrocarbon plume. We hypothesized that hydrogeological setting would impose a detectable spatial constraint on community turnover, but that functional organization and biodegradation potential would show stronger associations with community variation. We further hypothesized that high attenuation potential and high taxonomic-functional diversity would be only partly coincident across the monitoring network.

## 2 Materials and methods

### 2.1 Study site, hydrogeological setting, and groundwater sampling

Groundwater sampling was conducted in April 2025 at an oil depot in Qufu city, Shandong Province, China (117°02′14.16″E, 35°35′22.33″N). The depot was established in 1990 and occupies approximately 128,538 m^2^. A preliminary soil and groundwater investigation documented petroleum-related contamination at the site. Some investigated groundwater locations exceeded the applicable screening value for petroleum hydrocarbons (C10-C40), and benzene exceeded the Chinese Class III groundwater-quality limit. For the present study, groundwater was collected from 15 monitoring wells distributed across the depot (W1-W15; Figure 1A). Groundwater-level measurements from the monitoring-well network were used to construct a site-scale interpolated groundwater-level surface. Detailed information on monitoring-well construction and groundwater sampling is provided in the Supplementary methods.

**Figure 1:**
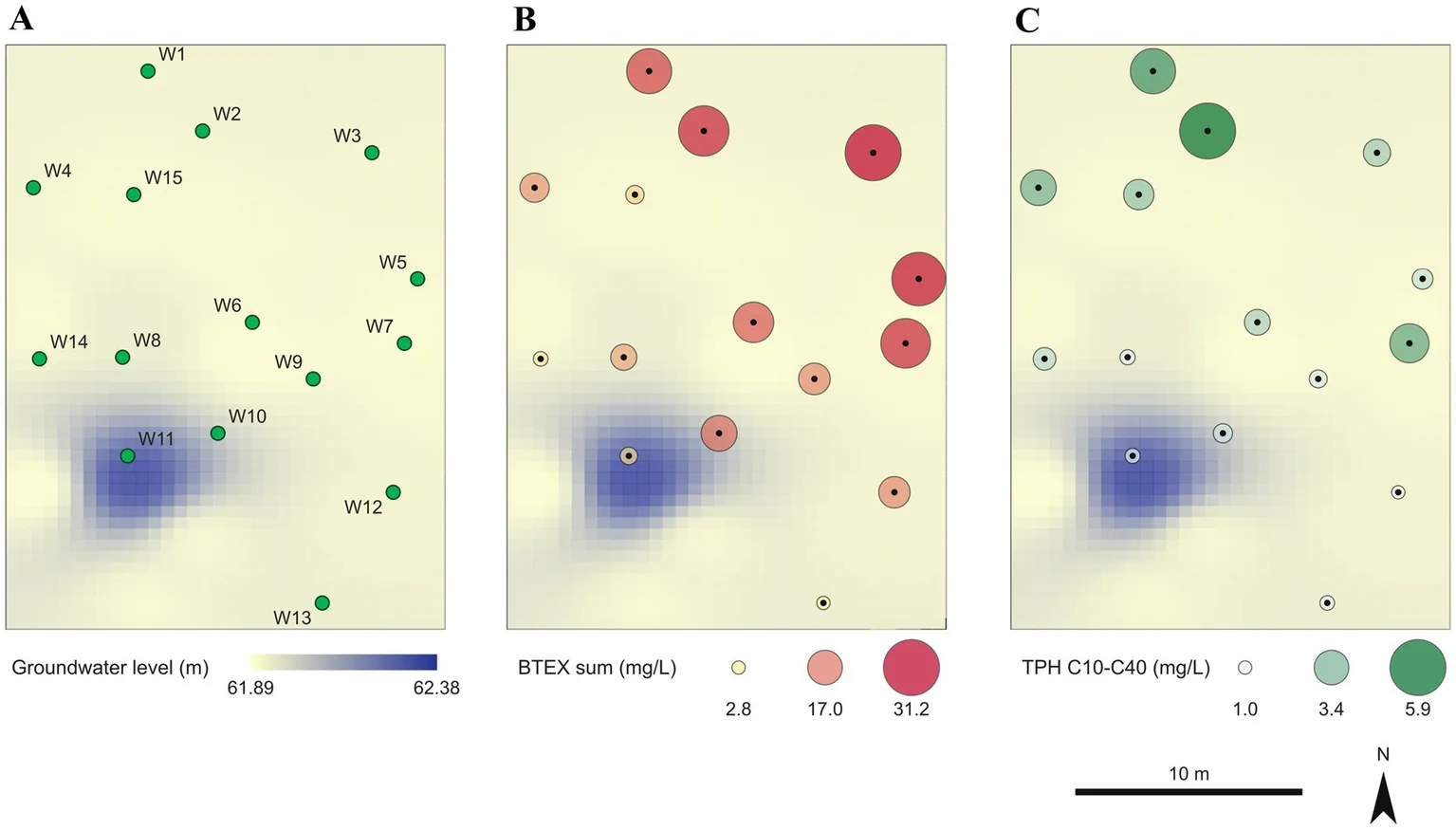
Spatial distribution of groundwater levels and petroleum hydrocarbons across the monitoring network. **(A)** Groundwater-elevation surface interpolated from well measurements using inverse-distance weighting, with monitoring wells W1–W15 indicated. **(B)** Sum concentrations of benzene, toluene, ethylbenzene, and xylenes (BTEX; mg·L^−1^). **(C)** Total petroleum hydrocarbons in the C10-C40 fraction (TPH C10-C40; mg·L^−1^). In **(B,C)**, symbol area and color intensity increase with concentration; black central dots identify well locations. The north arrow and 10-m scale bar apply to all panels.

### 2.2 Hydrochemical and petroleum-hydrocarbon measurements

Groundwater samples were analyzed for petroleum hydrocarbons and hydrochemical variables, including benzene, toluene, ethylbenzene, *m,p*-xylene, *o*-xylene, total petroleum hydrocarbons in the C10-C40 fraction (TPH C10-C40), pH, oxidation–reduction potential (ORP), nitrate, and total organic carbon (TOC). Benzene, toluene, ethylbenzene, *m,p*-xylene, and *o*-xylene were quantified using purge-and-trap gas chromatography–mass spectrometry in accordance with HJ 639–2012 (Ministry of Ecology and Environment of the People's Republic of China, 2012). Total BTEX concentration was calculated as the sum of these five target analytes. TPH C10-C40 was determined by gas chromatography following HJ 894–2017 (Ministry of Ecology and Environment of the People's Republic of China, 2017). The pH was determined by the electrode method according to HJ 1147–2020 (Ministry of Ecology and Environment of the People's Republic of China, 2020a). ORP was measured in accordance with the *Technical Specification for Environmental Monitoring of Groundwater* (HJ 164-2020; Ministry of Ecology and Environment of the People's Republic of China, 2020b) and reported in mV. Nitrate was determined by ion chromatography following HJ 84-2016 (Ministry of Ecology and Environment of the People's Republic of China, 2016), and TOC was measured by combustion oxidation followed by non-dispersive infrared absorption according to HJ 501-2009 (Ministry of Ecology and Environment of the People's Republic of China, 2009). The standard-method detection limits and lower limits of determination for the target analytes are provided in Supplementary Table S1, and the hydrochemical and petroleum-hydrocarbon measurement results for all monitoring wells are provided in Supplementary Table S2.

### 2.3 Metagenomic sequencing and annotation

Total genomic DNA was extracted from groundwater samples using the E.Z.N.A. Soil DNA Kit (Omega Bio-tek, Norcross, GA, USA). DNA concentration and purity were assessed using a TBS-380 fluorometer and NanoDrop 2000 spectrophotometer, respectively, and DNA integrity was checked by 1% agarose-gel electrophoresis. Qualified DNA was fragmented to approximately 500 bp using a Covaris M220 ultrasonicator (Gene Company Ltd., Hong Kong, China). Paired-end sequencing libraries were prepared with the NEXTflex Rapid DNA-Seq Kit (Bioo Scientific, Austin, TX, USA) and sequenced on an Illumina NovaSeq at Majorbio Bio-Pharm Technology Co., Ltd. (Shanghai, China).

Raw reads were inspected using SeqKit v0.15.0 and processed with Trimmomatic v0.39 to remove adapter sequences and low-quality reads (Bolger et al., 2014). The resulting high-quality reads were used for taxonomic and functional analyses. Taxonomic classification was performed with Kraken2 v2.1.2 against the MiniKraken2_v2_8GB_201904 database (Wood et al., 2019), and taxon abundances were re-estimated using Bracken (Lu et al., 2017). High-quality reads were assembled *de novo* using MEGAHIT v1.2.9; contigs shorter than 500 bp were excluded from subsequent analyses (Li et al., 2015). Open reading frames were predicted from assembled contigs using Prokka v1.14.6 (Seemann, 2014). A non-redundant gene catalog was generated with MMseqs2 v13.45111 at 95% sequence identity (Steinegger and Söding, 2017). Quality-controlled reads were mapped to this catalog using coverM v0.6.1 to quantify gene abundance in each sample. Functional annotation of the non-redundant gene catalog was performed using KofamScan v1.3.0 against the Kyoto Encyclopedia of Genes and Genomes (KEGG) database. KO assignments were retained only when they were marked by KofamScan as exceeding the predefined KO specific adaptive score threshold and had an E value no greater than 10^−5^. The assignments were subsequently summarized according to KEGG functional hierarchies and pathways (Kanehisa and Goto, 2000; Aramaki et al., 2020). The resulting taxonomic profiles, gene-abundance matrix, KEGG Ortholog annotations, and pathway-level functional profiles were used in the downstream community, functional-module, and biodegradation-potential analyses.

### 2.4 Spatial characterization and attenuation indices assessment

Pairwise Euclidean distances among the 15 monitoring wells were calculated from their locations and used to characterize site-scale spatial separation. The pollution-pressure index was calculated from z-standardized log-transformed total BTEX and TPH C10-C40 concentrations. A redox-resource-context score combined min-max normalized ORP, nitrate, and TOC. The biological-response index integrated degradation-associated genera, selected degradation pathways, and the redox-resource context. NAPI was calculated as the mean of min-max normalized pollution pressure, biological response, degradation-associated genus abundance, degradation-pathway abundance, and redox-resource context. Higher NAPI values were interpreted as stronger relative site-specific attenuation potential, reflecting greater joint representation of contaminant pressure, biological response, degradation-associated microbial features, and redox-resource context. Degradation-associated taxonomic-functional diversity was quantified using six components representing the richness and Shannon diversity of degradation-associated genera, pathways, and KEGG Orthologs (KOs). The ten selected pathways (Supplementary Table S3) comprised a nonredundant reference set of 350 KOs, of which 210 were detected in the study gene catalog and used for the KO-level diversity components (Supplementary Table S4). The complete set of detected KOs, together with gene symbols/products and associated pathways, is provided in Supplementary Table S5.

Each of the six components was min-max normalized and given equal weight in the composite taxonomic-functional diversity index. Degradation-associated functional diversity was calculated separately using the corresponding pathway- and KO-level richness and Shannon-diversity components, thereby excluding the genus-level metrics. Taxonomic and functional abundance profiles were normalized before calculation of the diversity-based components. Because richness depends on feature detection, normalization of relative abundance does not fully eliminate the potential influence of sequencing depth on richness estimates. The richness-based components and resulting composite indices were therefore interpreted as comparative site-level measures rather than sequencing-depth-independent estimates of absolute diversity. Operational attenuation states were assigned using distribution-based rules for pollution pressure, biological response, NAPI, and the pressure-response mismatch index. The latter was calculated as the standardized biological-response index minus the standardized pollution-pressure index. Active attenuation under high pressure was assigned when both pollution pressure and NAPI were at or above their upper tertiles. High pressure with insufficient response was assigned when pollution pressure was at or above its upper tertile but biological response remained below its upper tertile. Residual/recovery with legacy response was assigned when pollution pressure was below its lower tertile and the pressure-response mismatch was positive, whereas low-impact/background conditions were assigned when both pollution pressure and biological response were below their lower tertiles. Wells not meeting these criteria were classified as transition attenuation. Full component definitions, transformations, decision rules, and weighting-scenario analyses are provided in the Supplementary methods. Well-level values of the composite indices and the resulting operational attenuation-state classifications are summarized in Supplementary Table S6.

### 2.5 Statistical analysis

Statistical analyses were conducted using custom Python scripts in Python 3.12.13, with NumPy 2.3.5 and pandas 3.0.1. Community beta diversity was quantified from genus-level relative-abundance matrices using Bray–Curtis dissimilarity. Species-level analyses were conducted as supplementary analyses. Principal coordinates analysis (PCoA) was used to visualize community compositional variation. Permutational multivariate analysis of variance (PERMANOVA; 999 permutations) was used to test community differences among pollution groups, plume-position groups, response-pressure classes, and operational attenuation states.

Environmental, biodegradation-potential, taxonomic-functional diversity distance, and functional-module distance matrices were calculated as Euclidean distances from z-standardized variables or module scores. The genera and KEGG pathways comprising the predefined functional modules are listed in Supplementary Table S7. Mantel tests (999 permutations) evaluated associations between genus-level community dissimilarity and each explanatory distance matrix. Partial Mantel tests assessed the association between community dissimilarity and biodegradation-potential distance while controlling for estimated spatial distance.

Exploratory associations among taxa, functional modules, and operational indices were evaluated using Spearman correlation analyses with 999-permutation testing. Associations between measured geochemical variables (pH, ORP, nitrate, and TOC) and metagenome-derived degradation potential were additionally evaluated using two-sided Spearman permutation tests with 999 permutations. Primary geochemistry-function tests considered aggregate degradation-pathway abundance and degradation-associated functional diversity, whereas pathway-specific analyses considered the 10 selected degradation-associated KEGG pathways individually. For feature-level correlation screens, including taxon-module, taxon-environment, KO-environment, and pathway-environment associations, permutation *p*-values were adjusted using the Benjamini-Hochberg false-discovery-rate procedure within each corresponding family of related tests. Benjamini-Hochberg correction was also applied separately to the primary genus-level Mantel family and the primary genus-level PERMANOVA family. The partial Mantel test of biodegradation-potential distance while controlling for spatial distance was specified *a priori* and evaluated as a single test without multiple-testing correction. The robustness of well-level NAPI rankings was evaluated across alternative weighting scenarios. Nominal permutation *p*-values and Benjamini-Hochberg-adjusted q values are reported where applicable, and FDR-adjusted *q* < 0.05 was considered statistically significant for corrected test families.

## 3 Results

### 3.1 Hydrogeological setting and hydrocarbon heterogeneity

Fifteen groundwater monitoring wells (W1-W15) were distributed across the study area (Figure 1). Measured groundwater elevations ranged from 61.890 to 62.382 m. The interpolated groundwater-level surface showed a localized high-level zone superimposed on otherwise relatively small differences among wells, indicating hydrogeological heterogeneity at the scale of the monitoring network (Figure 1A).

Petroleum hydrocarbons displayed pronounced between-well variability. Total BTEX concentrations ranged from 2.77 to 31.21 mg·L^−1^ (median, 15.00 mg·L^−1^), with the highest value at W3 (31.21 mg·L^−1^). Elevated concentrations were also observed at W5 (29.82 mg·L^−1^), W2 (27.56 mg·L^−1^), W7 (27.02 mg·L^−1^), W1 (23.85 mg·L^−1^), and W6 (20.87 mg·L^−1^) (Figure 1B). In contrast, TPH C10-C40 concentrations ranged from 1.00 to 5.88 mg·L^−1^ (median, 2.06 mg·L^−1^) and reached their maximum at W2 (5.88 mg·L^−1^), followed by W1 (4.66 mg·L^−1^), W7 (3.96 mg·L^−1^), and W4 (3.56 mg·L^−1^) (Figure 1C). The spatial distributions of BTEX and TPH C10-C40 were therefore not fully coincident. For example, W3 had the highest BTEX concentration but a moderate TPH C10-C40 concentration (2.60 mg·L^−1^), whereas W4 showed relatively low BTEX (13.36 mg·L^−1^) but elevated TPH C10-C40.

### 3.2 Spatial and functional correlates of community turnover

At the genus level, Bray–Curtis dissimilarity increased weakly but significantly with estimated inter-well distance (Mantel *r* = 0.240, *P* = 0.034; Figure 2A and Table 1). Thus, pairs of wells separated by greater estimated distances tended to harbor more dissimilar communities. In contrast, the association between genus-community dissimilarity and environmental distance was not significant (Mantel *r* = 0.226, *p* = 0.107; Figure 2C). The 105 pairwise comparisons shown in Figure 2A provide a visualization of these distance relationships, whereas statistical inference was based on Mantel permutation tests.

**Figure 2:**
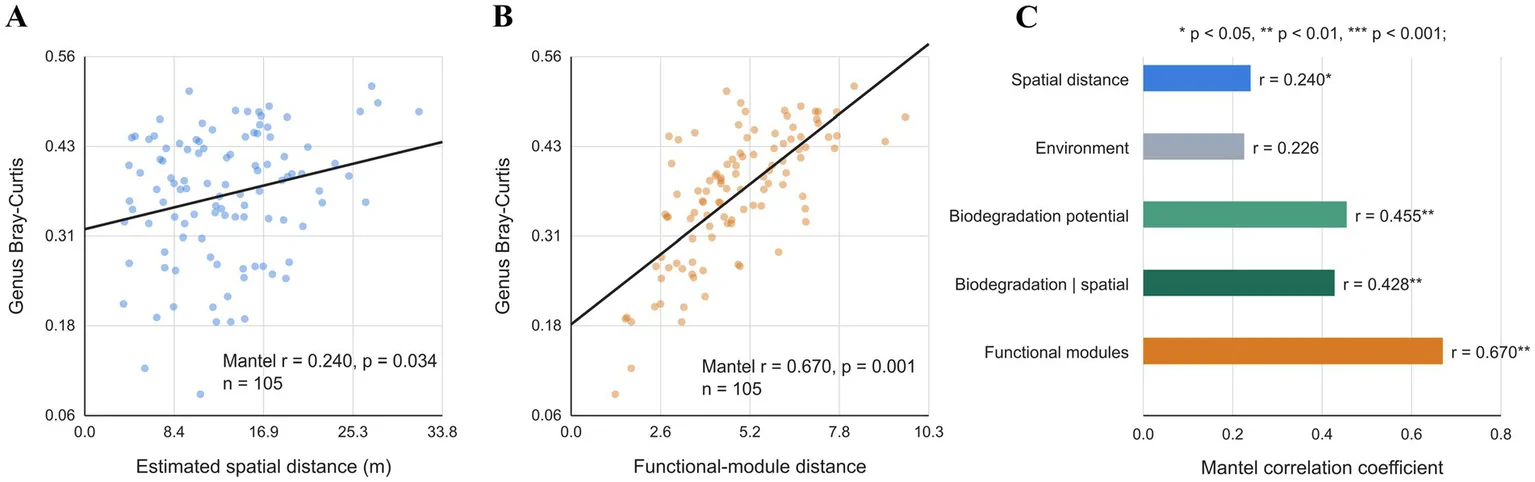
Associations of spatial separation, environmental variation, biodegradation potential, and functional organization with genus-level community dissimilarity. **(A)** Relationship between estimated pairwise spatial distance and genus-level Bray–Curtis dissimilarity. **(B)** Relationship between functional-module distance and genus-level Bray–Curtis dissimilarity. Each point represents a pairwise comparison between two monitoring wells (*n* = 105); solid lines represent least-squares fits. **(C)** Mantel correlations between genus-level community dissimilarity and spatial distance, environmental distance, biodegradation-potential distance, and functional-module distance. The partial Mantel correlation for biodegradation potential was calculated after controlling for spatial distance. Bars show Mantel correlation coefficients; asterisks indicate permutation significance (*p < 0.05, **p < 0.01, ***p < 0.001).

**Table 1:** Summary of statistical tests linking genus-level community variation with spatial distance, biodegradation potential, functional organization, and robustness metrics in petroleum-hydrocarbon-contaminated groundwater.

Analysis	Response/comparison	Predictor or grouping	Covariate controlled	Effect size/robustness metric	*p*-value	BH-adjusted *q* value
PERMANOVA	Genus community composition	Pollution group	–	*R*^2^ = 0.295	0.008	0.016
PERMANOVA	Genus community composition	Operational attenuation state	–	*R*^2^ = 0.354	0.008	0.016
Mantel test	Genus Bray–Curtis dissimilarity	Spatial distance	–	*r* = 0.240	0.034	0.0425
Mantel test	Genus Bray–Curtis dissimilarity	Functional-module distance	–	*r* = 0.670	0.001	0.005
Mantel test	Genus Bray–Curtis dissimilarity	Biodegradation-potential distance	–	*r* = 0.455	0.003	0.0075
Partial Mantel test	Genus Bray–Curtis dissimilarity	Biodegradation-potential distance	Spatial distance	*r* = 0.428	0.001	-
Mantel test	Taxonomic-functional diversity distance	Genus Bray–Curtis dissimilarity	–	*r* = 0.414	0.006	0.010
Robustness analysis	NAPI ranking	Top-five frequency across weighting scenarios	–	W1, W2, W3, W7 = 1.000	–	–
Robustness analysis	Taxonomic-functional diversity ranking	Top-five frequency across weighting scenarios	–	W13 = 1.000; W15 = 0.999; W12 = 0.890; W9 = 0.876; W14 = 0.820	–	–

*p*-values were obtained from 999 permutations. Benjamini-Hochberg false-discovery-rate correction was applied separately to the family of genus-level Mantel tests and the family of genus-level PERMANOVA tests. Corresponding adjusted q values are reported. The partial Mantel test of biodegradation-potential distance controlling for spatial distance was specified a priori and was evaluated as a single test. Therefore, no adjusted *q* value is reported.

Functional-module distance exhibited the strongest association with genus-level community dissimilarity among the tested distance matrices (Mantel *r* = 0.670, *p* = 0.001; Figures 2B,C and Table 1), substantially exceeding the association with spatial distance. Biodegradation-potential distance was also significantly associated with community dissimilarity (Mantel *r* = 0.455, *p* = 0.003). This relationship persisted after controlling for estimated spatial distance in a partial Mantel test (partial *r* = 0.428, *p* = 0.001), indicating that the observed correspondence between biodegradation potential and community turnover was retained after accounting for their spatial covariation. In addition, taxonomic-functional diversity distance was positively associated with genus-community dissimilarity (Mantel *r* = 0.414, *p* = 0.006; Table 1).

Direct well-level correlations further identified specific associations between measured geochemical conditions and degradation-associated functional potential. pH was positively correlated with aggregate degradation-pathway abundance (Spearman *ρ* = 0.760, permutation *p* = 0.002, BH-adjusted *q* = 0.016) and degradation-associated functional diversity (ρ = 0.687, *p* = 0.009, *q* = 0.036; Supplementary Table S8). At the individual-pathway level, pH was positively associated with ethylbenzene degradation (ρ = 0.821, *q* = 0.020), naphthalene degradation (ρ = 0.778, *q* = 0.040), and fluorobenzoate degradation (ρ = 0.810, *q* = 0.020) after BH correction. No aggregate-level associations with ORP, nitrate, or TOC remained significant after correction, and no pathway-specific associations with these variables passed the FDR threshold (Supplementary Figure S6 and Supplementary Table S8).

### 3.3 Community variation across operational attenuation states

The 15 wells were assigned to four operational attenuation states: active attenuation under high pressure (*n* = 5), transition attenuation zone (*n* = 5), residual/recovery with legacy response (*n* = 3), and low-impact/background (*n* = 2). Genus-level PCoA resolved 30.4 and 22.1% of the total Bray-Curtis variation along PCoA1 and PCoA2, respectively (Figure 3A). Wells classified as active attenuation under high pressure were concentrated at negative PCoA1 values, whereas residual/recovery and low-impact/background wells were located predominantly at positive PCoA1 values. Transition-zone wells occupied intermediate and broader ordination positions, overlapping partly with the other states.

**Figure 3:**
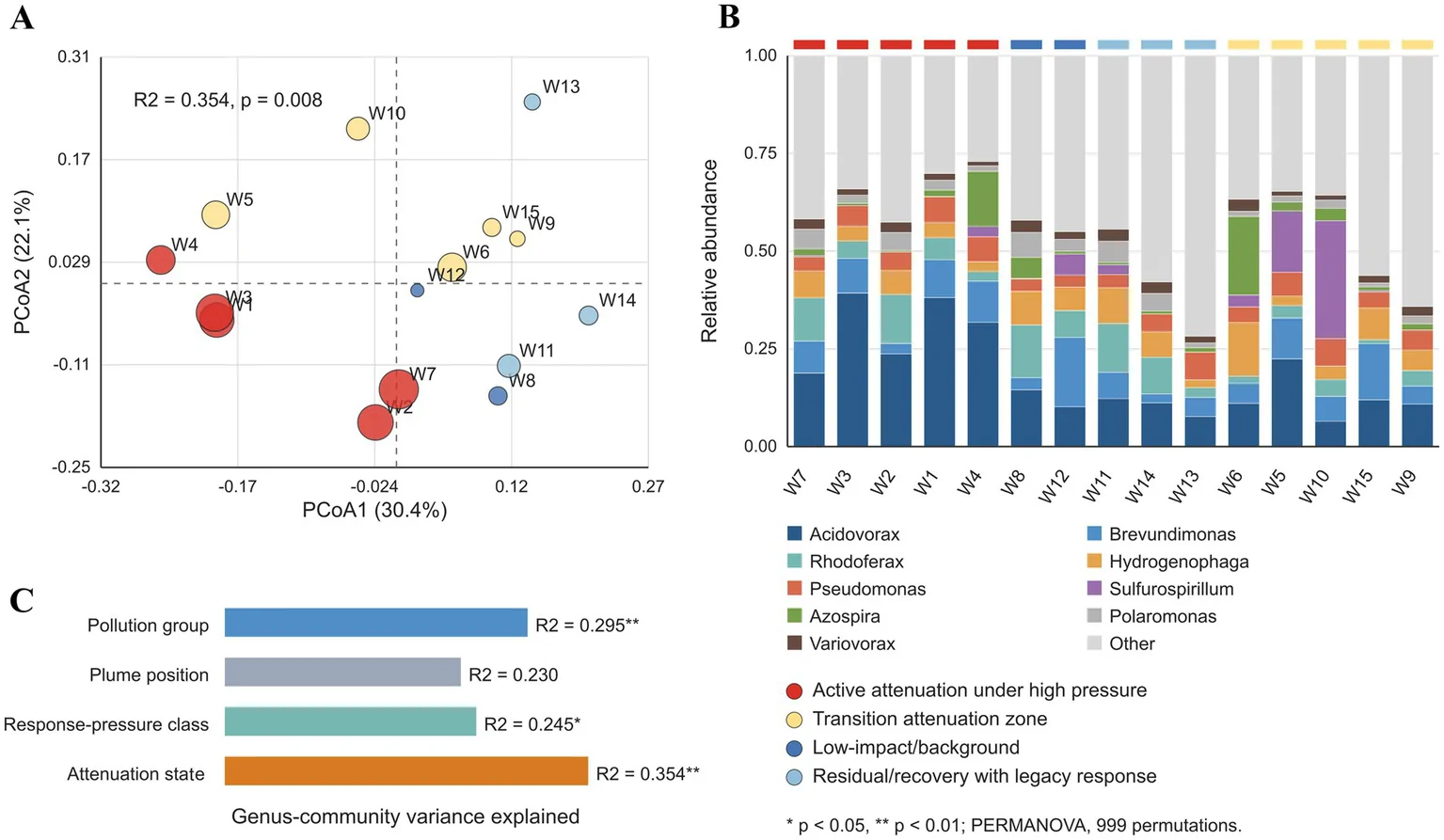
Genus-level community variation across pollution and operational attenuation categories. **(A)** Principal coordinates analysis (PCoA) of genus-level Bray-Curtis dissimilarities among the 15 monitoring wells. Colors indicate operational attenuation state, and point size is proportional to the natural attenuation potential index (NAPI). The displayed *R*^2^ and *p* value were obtained from PERMANOVA for attenuation state. **(B)** Relative abundances of the nine dominant genera, with the remaining genera grouped as other. Wells are ordered by operational attenuation state. The colored strip above the bars denotes the corresponding state. **(C)** Genus-community variation explained by four categorical frameworks, quantified as PERMANOVA *R*^2^ values: pollution group, plume position, response-pressure class, and operational attenuation state. *p < 0.05 and **p < 0.01, based on 999 permutations.

PERMANOVA confirmed that operational attenuation state was significantly associated with genus-level community composition (*R*^2^ = 0.354, *p* = 0.008; Figures 3A,C and Table 1; species-level results are provided in Supplementary Table S9). Among the tested categorical frameworks, attenuation state explained the largest proportion of community variation. Pollution group also explained a significant proportion of variation (*R*^2^ = 0.295, *p* = 0.008), whereas the response-pressure class showed a smaller but significant association (*R*^2^ = 0.245, *p* = 0.018). Plume position explained 23.0% of the variation but was not significant at *p* < 0.05 (*p* = 0.056; Figure 3C). All significant primary genus-level Mantel and PERMANOVA results remained significant after Benjamini-Hochberg correction within their respective test families (Table 1).

The relative abundances of dominant genera varied across operational attenuation states (Figure 3B). *Acidovorax* was most abundant in the active-attenuation group, with a mean relative abundance of 30.4%, compared with 12.6% in the transition zone and 10.4% in the residual/recovery group. The transition zone showed a higher mean relative abundance of *Sulfurospirillum* (9.8%) than the active-attenuation (0.6%), residual/recovery (0.8%), or low-impact/background (2.8%) groups. In contrast, *Rhodoferax* was relatively more abundant in the low-impact/background group (10.2%) than in the transition zone (2.8%). These compositional shifts were consistent with the state-associated separation observed in the ordination and PERMANOVA analyses.

### 3.4 Attenuation potential and taxonomic-functional diversity across wells

The pressure-response landscape showed substantial variation among wells (Figure 4A). Wells W1, W2, W3, and W7 combined elevated pollution-pressure and biological-response indices and were classified as active attenuation under high pressure. However, the two indices were not uniformly aligned across the monitoring network. The pressure-response mismatch index ranged from −1.062 at W9 to 1.457 at W11 (Figure 4C). W2 showed high pollution pressure but a comparatively lower biological response, resulting in a negative mismatch value (−0.816), whereas W7 and W3 had positive mismatch values (0.621 and 0.766, respectively). In contrast, residual/recovery wells W11, W13, and W14 showed positive mismatch values despite low pollution-pressure indices.

**Figure 4:**
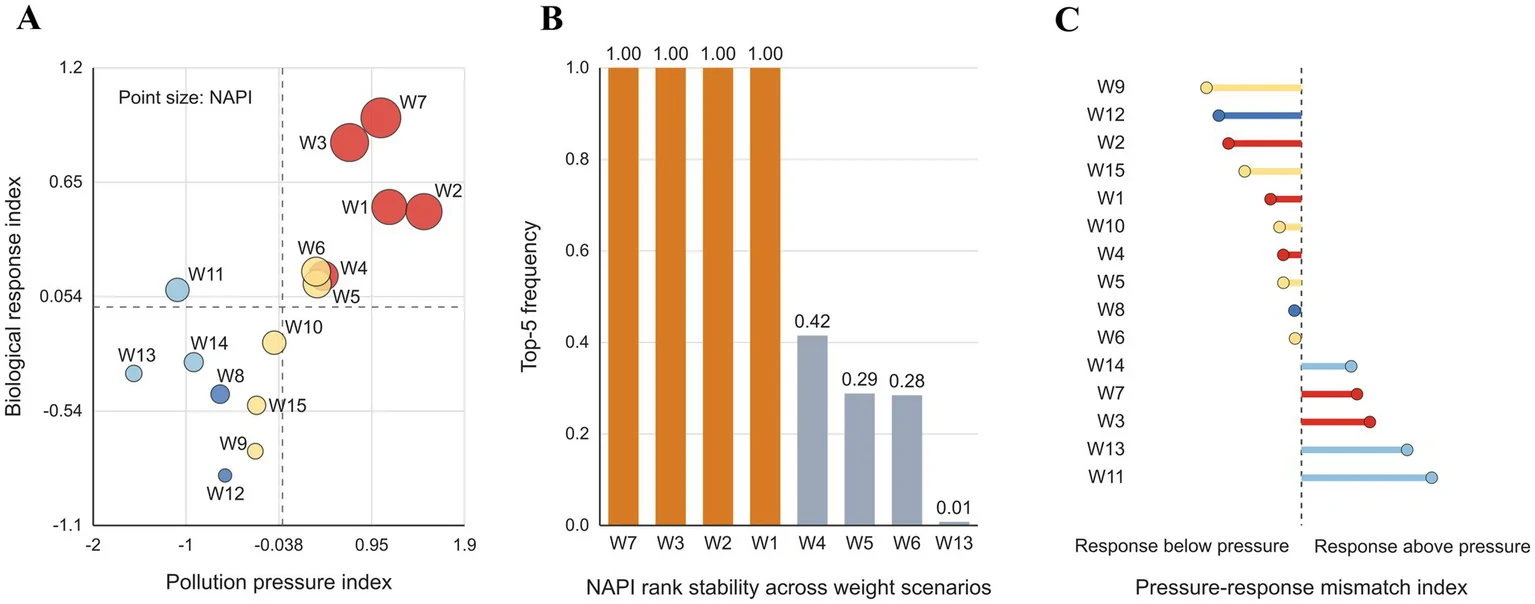
Comparison of pollution pressure, biological response, and natural attenuation potential across monitoring wells. **(A)** Wells projected onto standardized pollution-pressure and biological-response indices. Colors indicate operational attenuation state, and point size is proportional to the natural attenuation potential index (NAPI). Dashed lines denote zero values of the standardized indices. **(B)** Stability of well-level NAPI rankings across alternative weighting scenarios, expressed as the frequency with which each well ranked among the top five; the eight wells with the highest frequencies are shown. **(C)** Well-specific pressure-response mismatch, calculated as the standardized biological-response index minus the standardized pollution-pressure index. Negative values indicate biological response below pollution pressure, whereas positive values indicate biological response above pollution pressure. Colors indicate attenuation state.

W7, W3, W2, and W1 were identified as robust high-NAPI wells because they ranked among the top five wells in every alternative NAPI weighting scenario (top-five frequency = 1.00; Figure 4B and Table 1). These wells had NAPI values of 0.857, 0.812, 0.768, and 0.749, respectively. The next-ranked wells were less stable. W4 ranked in the top five in 41.5% of weighting scenarios, whereas W5 and W6 did so in 28.9 and 28.5% of scenarios, respectively. Thus, the identification of W1, W2, W3, and W7 as high-NAPI wells was insensitive to the weighting scheme used to construct the index.

The KO-level diversity components were based on the 210 detected degradation-associated KOs listed in Supplementary Tables S4, S5. Patterns of degradation-associated taxonomic-functional diversity differed from those of NAPI. The highest taxonomic-functional-diversity scores occurred at W13 (0.920) and W15 (0.766), followed by W9 (0.524), W12 (0.517), and W14 (0.515). These wells occupied partly distinct positions within the monitoring network from the robust high-NAPI wells. Rankings of taxonomic-functional diversity were also stable across weighting scenarios. W13, W15, W12, W9, and W14 ranked among the top five in 100.0, 99.9, 89.0, 87.6, and 82.0% of scenarios, respectively (Supplementary Table S10). Therefore, wells with high current attenuation potential did not generally coincide with those showing high degradation-associated taxonomic-functional diversity.

### 3.5 Functional-module profiles and taxon-module associations

The four functional modules displayed contrasting profiles across operational attenuation states (Figure 5B). The aromatic-hydrocarbon response module had its highest mean *z*-standardized score in the active-attenuation group (0.978) and its lowest value in the transition zone (−0.769). In contrast, the plume-margin redox-transition module was higher in the transition (0.205) and residual/recovery (0.243) groups than in the active-attenuation (−0.239) and low-impact/background (−0.281) groups. The metagenome-derived anaerobic terminal-process module also showed higher scores in the transition (0.156) and residual/recovery (0.230) groups. The PAH/naphthalene residual module showed the most distinct state profile, with a high mean score in the residual/recovery group (1.047) and negative mean scores in the active-attenuation (−0.131), transition (−0.414), and low-impact/background (−0.208) groups.

**Figure 5:**
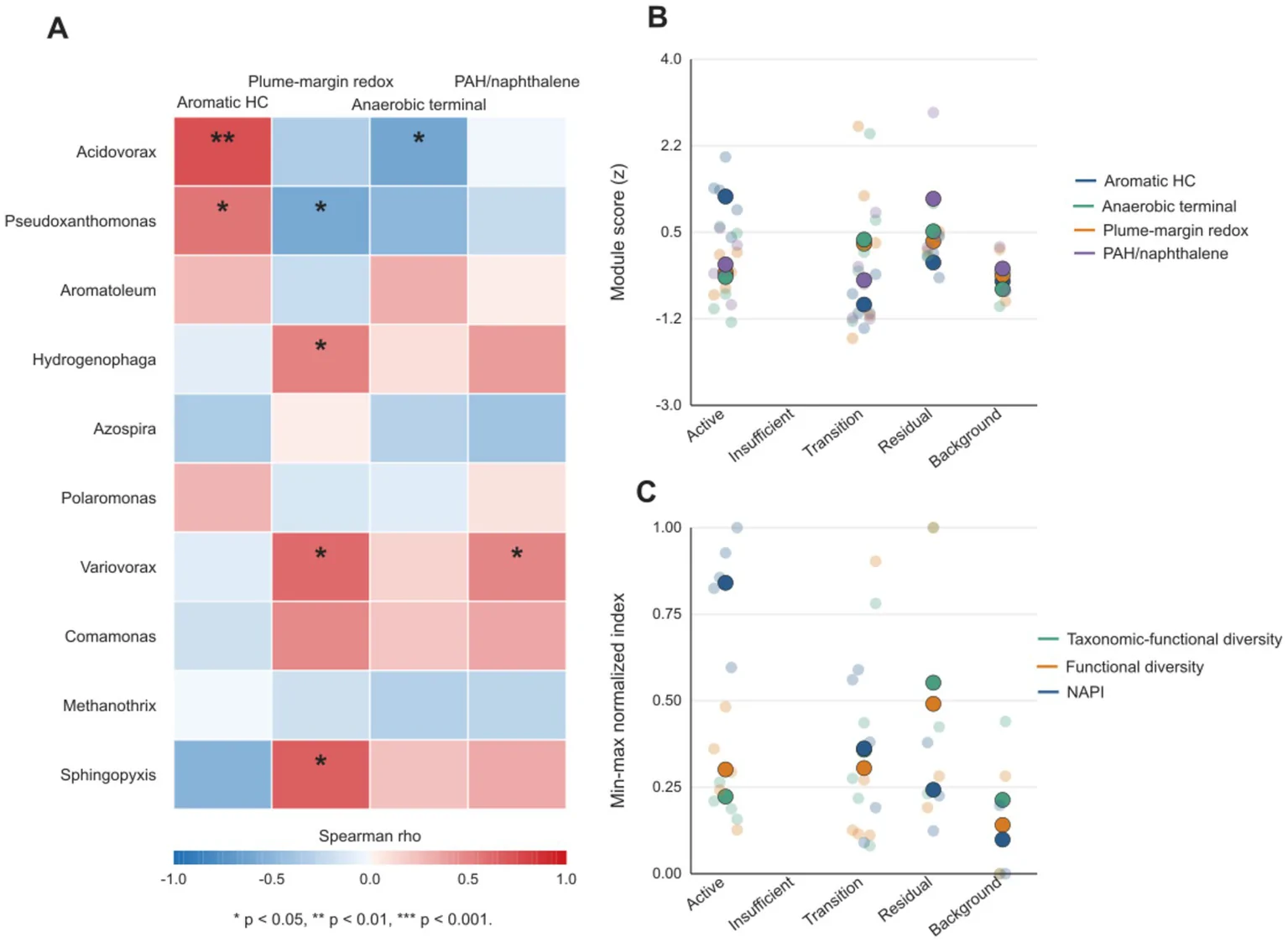
Relationships between hydrocarbon-associated genera, functional modules, and attenuation-state profiles. **(A)** Spearman correlations between the relative abundances of hydrocarbon-associated genera and four functional-module scores across the 15 monitoring wells. Cell colors represent Spearman correlation coefficients. Asterisks denote nominal significance from two-sided permutation tests (999 permutations; *p < 0.05, **p < 0.01, ***p < 0.001). No association remained significant after false-discovery-rate correction (*q* < 0.05). **(B)** Z-standardized scores of aromatic-hydrocarbon, anaerobic-terminal, plume-margin-redox, and PAH/naphthalene functional modules across operational attenuation states. Transparent circles represent individual wells, whereas solid circles and connecting lines represent state means. **(C)** Profiles of taxonomic-functional diversity, functional diversity, and the natural attenuation potential index (NAPI) across operational attenuation states. Each index was min-max normalized across all 15 wells. Transparent circles represent individual wells, and solid circles and connecting lines represent state means.

The three composite indices likewise showed non-identical state patterns (Figure 5C). Mean NAPI was highest in the active-attenuation group (0.759), followed by the transition group (0.465), residual/recovery group (0.392), and low-impact/background group (0.304). In contrast, taxonomic-functional diversity and functional diversity were highest in the residual/recovery group, with mean values of 0.605 and 0.560, respectively. The active-attenuation group had lower mean taxonomic-functional diversity (0.374) and functional diversity (0.410) despite its high NAPI. Thus, the attenuation-state profiles of current attenuation potential, taxonomic-functional diversity, and functional diversity were not congruent.

At the individual genus-module level, several nominal correlations were observed (Figure 5A). The strongest correlation was between *Acidovorax* and the aromatic-hydrocarbon response module (Spearman rho = 0.746, *p* = 0.002). Nominal associations were also detected between *Acidovorax* and the anaerobic terminal-process module (rho = −0.611, *p* = 0.011), *Sphingopyxis* and the plume-margin redox-transition module (rho = 0.675, *p* = 0.011), and *Variovorax* and the plume-margin redox-transition module (rho = 0.625, *p* = 0.013). However, none of the 40 tested genus-module associations remained significant after Benjamini-Hochberg false-discovery-rate correction (Supplementary Table S11).

## 4 Discussion

### 4.1 Hydrogeological constraints on microbial turnover

Groundwater microbial communities are shaped by both hydrological transport and local environmental selection. In the present study, genus-level Bray–Curtis dissimilarity increased significantly with estimated inter-well distance (Mantel *r* = 0.240, *p* = 0.034). Together with the heterogeneous groundwater-level field and non-uniform hydrocarbon distributions, this result indicates that microbial turnover was spatially structured within the monitoring network. Spatially structured microbial populations have also been reported along petroleum-contaminant plumes, where groundwater movement, aquifer heterogeneity, and contaminant distribution jointly influence the occurrence of microbial populations (Bekins et al., 2001). More recent groundwater studies likewise indicate that microbiological patterns can provide complementary information on subsurface connectivity and hydrogeochemical heterogeneity (Acciardo et al., 2025).

The spatial association observed here was nevertheless modest relative to the relationship between community dissimilarity and functional-module distance (*r* = 0.670). Physical separation therefore represented a constraint on community turnover rather than a sufficient explanation for it. Wells located farther apart tended to contain more dissimilar communities, but spatial distance alone did not capture the substantial variation associated with biodegradation potential, functional organization, and operational attenuation state. This distinction is important because a purely distance-based interpretation would understate the ecological differentiation evident across the hydrocarbon-impacted groundwater system.

It should be noted that pairwise Euclidean distance represents geographic separation rather than groundwater flow paths or direct hydraulic connectivity, and the cross-sectional design cannot distinguish dispersal limitation from transport along connected flow paths. Unmeasured aquifer properties and geochemical variables may also covary with well position and contribute to the observed community and functional patterns. Accordingly, the stronger functional associations indicate that spatial separation alone was not a sufficient explanation, but do not exclude hydrological or environmental controls not represented in the dataset.

### 4.2 Community-function coupling across attenuation states

The strongest association detected in this study was between genus-level community dissimilarity and functional-module distance (Mantel *r* = 0.670, *p* = 0.001), which was substantially greater than the association with estimated spatial distance (*r* = 0.240). Biodegradation-potential distance was also associated with community turnover (*r* = 0.455, *p* = 0.003), and this relationship persisted after controlling for spatial distance (partial Mantel *r* = 0.428, *p* = 0.001). These results indicate that the spatial reorganization of the groundwater microbiome was accompanied by changes in functional organization relevant to hydrocarbon transformation, rather than reflecting physical separation alone. In contrast, the measured environmental-distance matrix was not significantly associated with community dissimilarity (*r* = 0.226, *p* = 0.107), suggesting that the integrated functional signal captured variation not represented by the measured physicochemical variables at the same multivariate scale.

The direct geochemistry-function analysis provided a more specific view of these relationships. pH was positively associated with both aggregate degradation-pathway abundance and degradation-associated functional diversity, and positive associations with ethylbenzene-, naphthalene-, and fluorobenzoate-degradation pathways remained significant after FDR correction. In contrast, ORP, nitrate, and TOC showed no FDR-significant associations with the aggregate functional metrics. These results indicate that some components of degradation-associated functional potential covaried with local geochemical conditions, particularly pH, but were not consistently explained by any single measured redox or resource variable. Given the cross-sectional design and the fact that metagenomic profiles represent encoded potential rather than activity, the observed pH associations should be interpreted as site-specific covariation rather than evidence of direct geochemical control of hydrocarbon degradation.

The descriptive distributions of several dominant genera were broadly compatible with their reported ecological characteristics. *Acidovorax* was most abundant in the active-attenuation state and showed a nominal positive correlation with the aromatic-hydrocarbon response module. This pattern is consistent with reports that some *Acidovorax* strains can degrade aromatic hydrocarbons, including phenanthrene, and possess corresponding catabolic gene clusters (Singleton et al., 2009). *Sulfurospirillum* was relatively enriched in the transition state. Members of this genus can couple nitrate reduction to the oxidation of organic substrates or sulfide and have been recovered from oil-associated environments (Hubert and Voordouw, 2007), making their occurrence under heterogeneous redox conditions ecologically plausible. However, the available hydrochemical data do not support assignment of a specific sulfur-cycling, iron-reducing, or hydrocarbon-transforming role. *Rhodoferax* was more abundant in the low-impact/background state. Although some *Rhodoferax* species are facultatively anaerobic Fe(III) reducers and related populations have been detected in petroleum-contaminated subsurface environments (Finneran et al., 2003; Allen et al., 2007), its distribution in this study does not support its use as a hydrocarbon-specific indicator and may instead reflect broader adaptation to freshwater subsurface conditions. Moreover, none of the genus–module correlations remained significant after false-discovery-rate correction, and genus-level taxonomic assignments cannot resolve strain-specific metabolic traits. These comparisons therefore provide ecological context rather than evidence that these genera performed the inferred transformations *in situ*.

The operational attenuation-state framework provided a complementary representation of this community-function coupling. Attenuation state explained 35.4% of genus-level compositional variation, exceeding the explanatory value of the simpler pollution-group classification (29.5%). The active-attenuation group was characterized by high aromatic-hydrocarbon response-module scores and elevated NAPI, whereas modules associated with redox transition, anaerobic terminal processes, and residual PAH/naphthalene degradation showed relatively higher metagenome-derived scores in transition or residual/recovery wells. These patterns indicate spatial differentiation in annotated microbial and functional potential across the monitoring network (Kachienga et al., 2018; Ning et al., 2024). However, they do not identify the dominant terminal electron-accepting process at individual wells.

The functional modules and operational attenuation states were constructed as integrated descriptors that include biological-response information. Their association with community composition is therefore not an independent causal validation of microbial succession. Rather, the results identify a coherent spatial configuration in which taxonomic turnover, functional-module profiles, and biodegradation-potential metrics covary. Because sampling was cross-sectional and metagenomics measures functional potential rather than gene expression or transformation rates, the observed pattern should not be interpreted as direct evidence of temporal succession or *in situ* biodegradation activity. Nevertheless, the persistence of the biodegradation-potential association after accounting for spatial distance supports the use of community and functional information alongside hydrogeological context when distinguishing attenuation states.

### 4.3 Attenuation potential and degradation-associated diversity

The high-NAPI wells were identified consistently across alternative weighting schemes. W7, W3, W2, and W1 ranked among the top five wells in every scenario and were all classified as active attenuation under high pressure. This robustness indicates that their high NAPI values were not solely an artifact of a particular weighting configuration. These wells combined relatively high pollution-pressure and biological-response indices, and therefore represent locations where hydrocarbon exposure co-occurred with a strong attenuation-associated biological response.

Taxonomic-functional diversity showed a different pattern. The highest and most robust diversity scores occurred at W13, W15, W12, W9, and W14, which were largely assigned to residual/recovery, transition, or low-impact/background conditions rather than to the active-attenuation group. At the state level, NAPI was highest under active attenuation, whereas taxonomic-functional diversity and functional diversity were highest in the residual/recovery group. Thus, the two metrics did not identify the same wells or the same attenuation states. This difference is expected because NAPI was designed to integrate current pollution pressure with biological-response information, whereas the taxonomic-functional diversity score was constructed from the richness and Shannon diversity of degradation-associated taxonomic and annotated functional features.

Because the taxonomic-functional diversity score incorporates the richness and Shannon diversity of degradation-associated genera, pathways, and KOs, higher scores reflect richer and more even taxonomic and annotated functional profiles. Thus, the relatively high scores observed in residual/recovery or lower-pressure wells cannot be attributed exclusively to retained functional capacity. They may also reflect reduced taxonomic dominance or greater feature detectability. In particular, although abundance normalization reduces the direct influence of library size on relative-abundance-based diversity metrics, it does not fully remove sequencing-depth effects on richness. The score should therefore be interpreted as a richness- and diversity-based proxy for taxonomic-functional diversity. Accordingly, NAPI and the taxonomic-functional diversity proxy should be interpreted as complementary dimensions of natural attenuation.

### 4.4 Monitoring implications and study limitations

The results support a groundwater-monitoring strategy that integrates hydrogeological position, contaminant chemistry, and metagenome-derived microbial indicators. Hydrocarbon concentrations alone identified wells under greater contaminant pressure, but did not capture the observed variation in community composition, functional-module profiles, or pressure-response balance. Similarly, spatial position provided relevant context but did not account for the stronger association between community turnover and functional organization. Monitoring designs should therefore include wells spanning the hydrogeological and contamination gradients, rather than relying solely on concentration maxima or geographic spacing. This integrated approach is consistent with groundwater studies showing that hydrochemical and microbiological data provide complementary evidence of groundwater impacts and subsurface heterogeneity (Acciardo et al., 2025; Bagwell et al., 2025).

The complementary behavior of NAPI and taxonomic-functional diversity has practical implications for indicator selection. High-NAPI wells, particularly W1, W2, W3, and W7, represent candidate locations for tracking metagenome-inferred pressure-response patterns. In contrast, wells with high taxonomic-functional diversity identify locations with a comparatively broad and even representation of degradation-associated taxa and encoded functions, even where current pressure-response coupling is less pronounced. A monitoring framework that includes both types of indicators could distinguish high-response locations from locations that retain potentially resilient functional capacity. However, neither index should be used alone as a direct measure of hydrocarbon degradation rate or remediation performance.

Several limitations constrain interpretation. On the one hand, the study comprised 15 wells sampled at one time point. It therefore resolves spatial covariation but cannot establish temporal succession, directional microbial transport, or temporal stability of the observed community and functional-potential states. On the other hand, shotgun metagenomics characterizes taxonomic composition and encoded functional potential, but does not quantify gene expression, enzymatic activity, or *in situ* hydrocarbon transformation rates. In addition, the measured hydrochemical variables were limited to pH, ORP, nitrate, and TOC. Consequently, the available hydrochemical data could not distinguish among aerobic respiration, nitrate reduction, iron reduction, sulfate reduction, methanogenesis, and methane oxidation at individual wells. The observed functional modules and composite indices should therefore be interpreted as evidence of potential rather than direct process measurements. It should also be noted that the possible influence of background contamination on the metagenomic profiles cannot be completely excluded because no DNA-extraction blanks were included. Future work should combine surveyed well coordinates, repeated seasonal sampling, high-resolution hydrochemical measurements, and direct activity-based approaches, such as metatranscriptomics, stable-isotope probing, or compound-specific isotope analysis. Such data would allow the present spatial associations to be tested against temporal dynamics and measured attenuation processes.

## 5 Conclusion

This study integrated hydrogeological context, petroleum-hydrocarbon concentrations, and shotgun-metagenomic data to examine microbial variation across 15 groundwater monitoring wells. Genus-level community turnover showed a significant but modest association with estimated spatial distance, indicating hydrogeological constraints on microbial distribution. However, community dissimilarity was more strongly associated with functional-module organization and biodegradation potential, including after controlling for spatial distance.

Operational attenuation states captured substantial variation in community composition and functional-module profiles. High metagenome-inferred natural attenuation potential was robustly identified in a subset of high-pressure wells, whereas high taxonomic-functional diversity occurred primarily in different wells, demonstrating that current attenuation potential and degradation-associated diversity are complementary rather than interchangeable indicators. These findings are based on a single sampling event and therefore describe spatial covariation rather than temporal dynamics. Moreover, the metagenomic profiles represent encoded functional potential and do not establish realized *in situ* metabolic activity or hydrocarbon degradation rates. Within these interpretive boundaries, integrating hydrogeological, chemical, taxonomic, and functional evidence can improve the identification and interpretation of heterogeneous attenuation states in petroleum-hydrocarbon-contaminated groundwater.

## Data Availability

All data supporting the findings of this study are included in the manuscript and its Supplementary Tables. The metadata associated with this study have been deposited in NCBI under BioProject accession number PRJNA1495749, with associated BioSample accessions SAMN61645234-SAMN61645248.
