# Supplementary material for: Functional organization outweighs spatial separation in structuring microbial communities across a petroleum-hydrocarbon groundwater plume
Source: Front Microbiol. 2026 Sep 16;17:1938677. doi: 10.3389/fmicb.2026.1938677 (PMC13624111; doi:10.3389/fmicb.2026.1938677)
Supplement: Supplementary file 1 [file Supplementary_file_1.DOCX]

Supplementary Material

*Functional organization outweighs spatial separation in structuring microbial communities across a petroleum-hydrocarbon groundwater plume*

# 1 Supplementary Methods

## 1.1 Monitoring-well construction and groundwater sampling

Fifteen groundwater monitoring wells were installed using a Geoprobe 7822DT drilling rig. Each well was completed with 53-mm-diameter rigid polyvinyl chloride (PVC) casing and screen. The casing sections were connected using mechanical snap-fit joints without adhesives, with the sections aligned along a common vertical axis. The well screens had 0.2-mm-wide openings, with drilled perforations no greater than 5 mm in diameter and spaced 10–20 mm apart. The screened interval extended across the target water-bearing interval, with its upper end above the groundwater level, and blank seamless PVC casing was installed from the top of the screen to above ground level. The annular materials consisted, from bottom to top, of a filter pack, sealing layer, and upper backfill. The filter pack consisted of clean, well-rounded quartz sand with a grain size of 1–2 mm, and bentonite was used as the sealing material.

Well development was initiated at least 8 h after installation and was performed using a bailer. Development continued until the extracted groundwater was visually clear and free of sediment and the monitored field parameters, including pH, electrical conductivity, turbidity, and temperature, had stabilized. Stabilization was defined as variation within ±10% over three consecutive measurements or, for turbidity, a value below 50 NTU.

Before groundwater sampling, each well was allowed to equilibrate for more than 24 h. The stabilized depth to groundwater below the top of the well casing was measured before purging and ranged approximately from 7 to 9 m among the wells. Each well was then purged using a bailer until three to four standing well volumes had been removed. During purging, the extracted groundwater was inspected for color, odor, and suspended material, and pH, dissolved oxygen, temperature, turbidity, electrical conductivity, and oxidation–reduction potential were monitored in the field. Purging was considered complete when successive measurements met the stabilization criteria.

Groundwater sampling was completed within 2 h after purging. Before sample collection, the bailer was rinsed several times with deionized water and then three times with groundwater from the corresponding well. The bailer was lowered below the groundwater surface into the saturated screened interval. Because groundwater was collected using a bailer rather than a pump with a fixed intake, no single fixed intake depth was used. Water was transferred slowly along the inner wall of each sample container to minimize agitation, aeration, and bubble formation.

Samples for volatile organic compound analysis, including BTEX, were collected in pre-preserved 40-mL vials containing HCl. The vials were completely filled, and any remaining headspace was displaced before the caps were secured. Samples for semivolatile organic compounds and TPH C10–C40 analysis were collected separately in 1-L amber glass bottles. Separate aliquots were collected for the other hydrochemical analyses. For microbial analysis, a separate 4L groundwater sample was collected from each monitoring well. The entire 4L sample was filtered through a 0.22 μm pore size membrane filter (Millipore, Billerica, MA, USA).

Immediately after collection, all sample bottles were sealed, labeled, wrapped in protective foam, and placed in ice-packed coolers. The samples were protected from direct sunlight, elevated temperature, moisture, and acidic or alkaline vapors during transport. Additional cushioning material was placed inside the coolers to minimize agitation and prevent bottle breakage. Samples were transported to the laboratory under chilled conditions at approximately 4 °C.

## 1.2 Spatial characterization and composite indices

Relative positions of the 15 monitoring wells and the groundwater-level field were digitized from the available site map. Inter-well Euclidean distances were estimated from the map scale and were used only as site-scale descriptors of relative spatial separation. The pollution-pressure index was calculated as the mean z score of log1p-transformed total BTEX and TPH C10-C40 concentrations. A redox-resource-context score was calculated as the mean min-max normalized value of ORP, nitrate, and TOC. The biological-response index was the mean of z-standardized degradation-associated genus abundance, degradation-pathway abundance, and the redox-resource-context score.

The natural attenuation potential index (NAPI) was calculated as the mean of min-max normalized pollution pressure, biological response, degradation-associated genus abundance, degradation-pathway abundance, and redox-resource context. Taxonomic-functional diversity was calculated from the min-max normalized richness and Shannon diversity of degradation-associated genera, pathways, and KEGG Orthologs (KOs). Functional completeness was calculated from the corresponding pathway- and KO-level richness and Shannon-diversity metrics. These composite indices were exploratory, site-specific comparative descriptors and were not interpreted as direct measures of in situ degradation rates.

Operational attenuation states were assigned using distribution-based thresholds. Wells with pollution pressure and NAPI at or above the upper tertile were classified as active attenuation under high pressure. Wells with pollution pressure at or above the upper tertile but biological response below the upper tertile were classified as high pressure with insufficient response. Wells with pollution pressure below the lower tertile and a positive pressure-response mismatch were classified as residual/recovery with legacy response; wells with both pollution pressure and biological response below the lower tertile were classified as low impact/background. Remaining wells were classified as transition attenuation zone. The pressure-response mismatch index was calculated as the standardized biological-response index minus the standardized pollution-pressure index.

## 1.3 Functional modules and sensitivity analyses

Four functional modules were constructed from pre-specified hydrocarbon-associated genera and KEGG pathway categories (Table S3). Module scores were calculated from standardized component abundances. Sensitivity analyses recomputed NAPI and taxonomic-functional diversity rankings over alternative component-weighting scenarios. Ranking stability was summarized by the median, best, and worst rank and the frequency with which a well was included among the top three or top five wells. Taxon-module associations were considered exploratory; nominal permutation P values were adjusted across all tested genus-module pairs using the Benjamini-Hochberg procedure.

# 3 Supplementary Figures

Figure S1. Detailed petroleum-hydrocarbon and hydrochemical profiles across monitoring wells. Values are standardized by variable across the 15 wells (row z scores).

Figure S2. Species-level beta-diversity ordination. Principal coordinates analysis was based on species-level Bray-Curtis dissimilarity. Colors indicate the pollution group, and point size is proportional to total BTEX concentration. (Axis1 29.2%, Axis2 21.4%)

Figure S3. Well-level functional-module scores. Wells are ordered by operational attenuation state. Colors represent z-standardized functional-module scores, and the colored strip identifies the attenuation state.

Figure S4. Sensitivity of NAPI components and rankings across alternative weighting scenarios. Rows show summary statistics for individual wells; columns are ordered by the median NAPI rank across scenarios.

Figure S5. Sensitivity of taxonomic-functional diversity components and rankings across alternative weighting scenarios. Rows show summary statistics for individual wells; columns are ordered by the median taxonomic-functional diversity rank across scenarios.

Figure S6. Spearman correlations between degradation pathways and geochemical variables. Cell labels show rho; * indicates BH-adjusted q < 0.05 across 40 pathway-by-geochemistry tests.

# 5 Supplementary Tables

**Table S1. Standard-method detection limits and lower limits of determination for petroleum-hydrocarbon analytes.**

| **Analyte** | **Analytical method** | **Method detection limit (μg L^-1^)** | **Lower limit of determination (μg L^-1^)** |
| --- | --- | --- | --- |
| Benzene | HJ 639-2012 | 1.4 | 5.6 |
| Toluene |  | 1.4 | 2.6 |
| Ethylbenzene |  | 0.8 | 3.2 |
| *m,p*-Xylene |  | 2.2 | 8.8 |
| *o*-Xylene |  | 1.4 | 5.6 |
| TPH C10-C40 | HJ 894-2017 | 10 | 40 |

Note: The listed values are the standard-method limits specified in HJ 639-2012 and HJ 894-2017, rather than laboratory-specific limits established for the present analytical batch. The values for BTEX correspond to the full-scan acquisition mode specified in HJ 639-2012. For TPH C10–C40, the limits apply to a sample volume of 1,000 mL. The “<0.1 mg L^-1^” value in the original ethylbenzene dataset represents the laboratory reporting threshold and should not be interpreted as the method detection limit.

**Table S2. Petroleum-hydrocarbon concentrations and hydrochemical variables in groundwater samples.**

| **Well** | **Benzene (mg L-1)** | **Toluene (mg L-1)** | **Ethylbenzene (mg L-1)** | **m/p-Xylene (mg L-1)** | **o-Xylene (mg L-1)** | **BTEX (mg L-1)** | **TPH C10-C40 (mg L-1)** | **pH** | **ORP (mV)** | **Nitrate (mg L-1)** | **TOC (mg L-1)** |
| --- | --- | --- | --- | --- | --- | --- | --- | --- | --- | --- | --- |
| W1 | 3.797 | 15.353 | 0.718 | 2.850 | 1.133 | 23.851 | 4.660 | 7.333 | 111.0 | 106.0 | 348.0 |
| W2 | 4.243 | 17.279 | 0.862 | 3.767 | 1.407 | 27.558 | 5.880 | 7.347 | 75.000 | 107.0 | 228.0 |
| W3 | 3.640 | 20.461 | 1.022 | 4.544 | 1.540 | 31.207 | 2.600 | 7.353 | 151.0 | 107.0 | 292.0 |
| W4 | 4.309 | 5.668 | 0.837 | 2.066 | 0.477 | 13.357 | 3.560 | 7.287 | 17.000 | 115.0 | 276.0 |
| W5 | 3.816 | 19.439 | 0.994 | 4.247 | 1.324 | 29.820 | 1.860 | 7.217 | 80.000 | 113.0 | 457.0 |
| W6 | 3.402 | 13.293 | 0.674 | 2.484 | 1.019 | 20.872 | 2.420 | 7.260 | 126.0 | 100.0 | 381.0 |
| W7 | 3.663 | 17.392 | 0.861 | 3.745 | 1.363 | 27.024 | 3.960 | 7.333 | 80.000 | 112.0 | 427.0 |
| W8 | 3.104 | 5.759 | 0.499 | 1.473 | 0.447 | 11.282 | 1.210 | 7.310 | 52.000 | 100.0 | 223.0 |
| W9 | 2.699 | 9.463 | 0.488 | 1.689 | 0.657 | 14.996 | 1.520 | 7.293 | 66.000 | 114.0 | 246.0 |
| W10 | 3.334 | 10.878 | 0.707 | 2.038 | 0.917 | 17.874 | 1.670 | 7.213 | 69.000 | 106.0 | 309.0 |
| W11 | 2.489 | 2.098 | 0.350 | 0.439 | 0.326 | 5.702 | 1.200 | 7.283 | 72.000 | 106.0 | 290.0 |
| W12 | 2.868 | 8.747 | 0.639 | 1.827 | 0.728 | 14.809 | 1.000 | 7.310 | 71.000 | 100.0 | 215.0 |
| W13 | 1.404 | 1.006 | 0.050 | 0.207 | 0.106 | 2.773 | 1.140 | 7.413 | 178.0 | 100.0 | 159.0 |
| W14 | 2.008 | 0.907 | 0.251 | 0.336 | 0.123 | 3.625 | 2.060 | 7.273 | 63.000 | 108.0 | 247.0 |
| W15 | 1.491 | 3.451 | 0.278 | 0.644 | 0.256 | 6.120 | 2.960 | 7.540 | 86.500 | 106.0 | 188.0 |

**Table S3. KEGG pathways included in the degradation-associated pathway set.**

| **Degradation-associated KEGG pathway** | |
| --- | --- |
| Benzoate degradation | Polycyclic aromatic hydrocarbon degradation |
| Xylene degradation | Aminobenzoate degradation |
| Toluene degradation | Nitrotoluene degradation |
| Ethylbenzene degradation | Styrene degradation |
| Naphthalene degradation | Fluorobenzoate degradation |

**Table S4. Coverage of the selected degradation-associated KEGG pathways in the study gene catalog.**

| **KEGG pathway** | **Reference unique KOs** | **Detected KOs** | **Undetected KOs** |
| --- | --- | --- | --- |
| Benzoate degradation | 116 | 89 | 27 |
| Xylene degradation | 34 | 28 | 6 |
| Toluene degradation | 49 | 32 | 17 |
| Ethylbenzene degradation | 16 | 8 | 8 |
| Naphthalene degradation | 28 | 12 | 16 |
| Polycyclic aromatic hydrocarbon degradation | 43 | 19 | 24 |
| Aminobenzoate degradation | 95 | 56 | 39 |
| Nitrotoluene degradation | 23 | 17 | 6 |
| Styrene degradation | 25 | 15 | 10 |
| Fluorobenzoate degradation | 18 | 10 | 8 |
| Nonredundant total | 350 | 210 | 140 |

**Table S5.** **KEGG Orthologs included in the degradation-associated KO set.**

| **KEGG Ortholog** | **Gene symbol/product** | **Associated pathway(s)** |
| --- | --- | --- |
| K00055 | E1.1.1.90; aryl-alcohol dehydrogenase [EC:1.1.1.90] | Xylene degradation; Toluene degradation |
| K00074 | paaH, hbd, fadB, mmgB; 3-hydroxybutyryl-CoA dehydrogenase [EC:1.1.1.157] | Benzoate degradation |
| K00121 | frmA, ADH5, adhC; S-(hydroxymethyl)glutathione dehydrogenase / alcohol dehydrogenase [EC:1.1.1.284 1.1.1.1] | Naphthalene degradation |
| K00141 | xylC; benzaldehyde dehydrogenase (NAD) [EC:1.2.1.28] | Xylene degradation; Toluene degradation; Aminobenzoate degradation |
| K00146 | feaB, tynC; phenylacetaldehyde dehydrogenase [EC:1.2.1.39] | Styrene degradation |
| K00169 | porA; pyruvate ferredoxin oxidoreductase alpha subunit [EC:1.2.7.1] | Nitrotoluene degradation |
| K00170 | porB; pyruvate ferredoxin oxidoreductase beta subunit [EC:1.2.7.1] | Nitrotoluene degradation |
| K00171 | porD; pyruvate ferredoxin oxidoreductase delta subunit [EC:1.2.7.1] | Nitrotoluene degradation |
| K00172 | porC, porG; pyruvate ferredoxin oxidoreductase gamma subunit [EC:1.2.7.1] | Nitrotoluene degradation |
| K00196 | cooF; anaerobic carbon-monoxide dehydrogenase iron sulfur subunit | Nitrotoluene degradation |
| K00198 | cooS, acsA; anaerobic carbon-monoxide dehydrogenase catalytic subunit [EC:1.2.7.4] | Nitrotoluene degradation |
| K00217 | E1.3.1.32; maleylacetate reductase [EC:1.3.1.32] | Benzoate degradation; Toluene degradation; Fluorobenzoate degradation |
| K00252 | GCDH, gcdH; glutaryl-CoA dehydrogenase [EC:1.3.8.6] | Benzoate degradation |
| K00446 | dmpB, xylE; catechol 2,3-dioxygenase [EC:1.13.11.2] | Benzoate degradation; Xylene degradation; Styrene degradation |
| K00448 | pcaG; protocatechuate 3,4-dioxygenase, alpha subunit [EC:1.13.11.3] | Benzoate degradation; Polycyclic aromatic hydrocarbon degradation |
| K00449 | pcaH; protocatechuate 3,4-dioxygenase, beta subunit [EC:1.13.11.3] | Benzoate degradation; Polycyclic aromatic hydrocarbon degradation |
| K00451 | HGD, hmgA; homogentisate 1,2-dioxygenase [EC:1.13.11.5] | Styrene degradation |
| K00480 | E1.14.13.1; salicylate hydroxylase [EC:1.14.13.1] | Naphthalene degradation; Polycyclic aromatic hydrocarbon degradation |
| K00481 | pobA; p-hydroxybenzoate 3-monooxygenase [EC:1.14.13.2] | Benzoate degradation |
| K00529 | hcaD; 3-phenylpropionate/trans-cinnamate dioxygenase ferredoxin reductase component [EC:1.18.1.3] | Benzoate degradation |
| K00626 | ACAT, atoB; acetyl-CoA C-acetyltransferase [EC:2.3.1.9] | Benzoate degradation |
| K00632 | fadA, fadI; acetyl-CoA acyltransferase [EC:2.3.1.16] | Benzoate degradation; Ethylbenzene degradation |
| K01026 | pct; propionate CoA-transferase [EC:2.8.3.1] | Styrene degradation |
| K01031 | pcaI; 3-oxoadipate CoA-transferase, alpha subunit [EC:2.8.3.6] | Benzoate degradation |
| K01032 | pcaJ; 3-oxoadipate CoA-transferase, beta subunit [EC:2.8.3.6] | Benzoate degradation |
| K01034 | atoD; acetate CoA/acetoacetate CoA-transferase alpha subunit [EC:2.8.3.8 2.8.3.9] | Aminobenzoate degradation |
| K01035 | atoA; acetate CoA/acetoacetate CoA-transferase beta subunit [EC:2.8.3.8 2.8.3.9] | Aminobenzoate degradation |
| K01039 | gctA; glutaconate CoA-transferase, subunit A [EC:2.8.3.12] | Styrene degradation |
| K01040 | gctB; glutaconate CoA-transferase, subunit B [EC:2.8.3.12] | Styrene degradation |
| K01055 | pcaD; 3-oxoadipate enol-lactonase [EC:3.1.1.24] | Benzoate degradation |
| K01061 | clcD; carboxymethylenebutenolidase [EC:3.1.1.45] | Toluene degradation; Fluorobenzoate degradation |
| K01075 | E3.1.2.23; 4-hydroxybenzoyl-CoA thioesterase [EC:3.1.2.23] | Benzoate degradation |
| K01426 | E3.5.1.4, amiE; amidase [EC:3.5.1.4] | Aminobenzoate degradation; Styrene degradation |
| K01501 | E3.5.5.1; nitrilase [EC:3.5.5.1] | Aminobenzoate degradation; Styrene degradation |
| K01512 | acyP; acylphosphatase [EC:3.6.1.7] | Aminobenzoate degradation |
| K01555 | FAH, fahA; fumarylacetoacetase [EC:3.7.1.2] | Styrene degradation |
| K01576 | mdlC; benzoylformate decarboxylase [EC:4.1.1.7] | Aminobenzoate degradation |
| K01607 | pcaC; 4-carboxymuconolactone decarboxylase [EC:4.1.1.44] | Benzoate degradation |
| K01612 | bsdC; vanillate/4-hydroxybenzoate decarboxylase subunit C [EC:4.1.1.- 4.1.1.61] | Aminobenzoate degradation |
| K01615 | gcdA; glutaconyl-CoA decarboxylase subunit alpha [EC:7.2.4.5] | Benzoate degradation |
| K01617 | dmpH, xylI, nahK; 2-oxo-3-hexenedioate decarboxylase [EC:4.1.1.77] | Benzoate degradation; Xylene degradation |
| K01666 | mhpE; 4-hydroxy 2-oxovalerate aldolase [EC:4.1.3.39] | Benzoate degradation; Xylene degradation |
| K01692 | paaF, echA; enoyl-CoA hydratase [EC:4.2.1.17] | Benzoate degradation; Aminobenzoate degradation |
| K01721 | nthA; nitrile hydratase subunit alpha [EC:4.2.1.84] | Aminobenzoate degradation; Styrene degradation; Fluorobenzoate degradation |
| K01781 | mdlA; mandelate racemase [EC:5.1.2.2] | Aminobenzoate degradation |
| K01782 | fadJ; 3-hydroxyacyl-CoA dehydrogenase / enoyl-CoA hydratase / 3-hydroxybutyryl-CoA epimerase [EC:1.1.1.35 4.2.1.17 5.1.2.3] | Benzoate degradation |
| K01800 | maiA, GSTZ1; maleylacetoacetate isomerase [EC:5.2.1.2] | Styrene degradation |
| K01821 | praC, xylH; 4-oxalocrotonate tautomerase [EC:5.3.2.6] | Benzoate degradation; Xylene degradation |
| K01825 | fadB; 3-hydroxyacyl-CoA dehydrogenase / enoyl-CoA hydratase / 3-hydroxybutyryl-CoA epimerase / enoyl-CoA isomerase [EC:1.1.1.35 4.2.1.17 5.1.2.3 5.3.3.8] | Benzoate degradation |
| K01856 | catB; muconate cycloisomerase [EC:5.5.1.1] | Benzoate degradation; Toluene degradation; Fluorobenzoate degradation |
| K01857 | pcaB; 3-carboxy-cis,cis-muconate cycloisomerase [EC:5.5.1.2] | Benzoate degradation |
| K01865 | hab; (hydroxyamino)benzene mutase [EC:5.4.4.1] | Aminobenzoate degradation |
| K02554 | mhpD; 2-keto-4-pentenoate hydratase [EC:4.2.1.80] | Benzoate degradation; Xylene degradation |
| K03186 | ubiX, bsdB, PAD1; flavin prenyltransferase [EC:2.5.1.129] | Aminobenzoate degradation |
| K03380 | E1.14.13.7; phenol 2-monooxygenase (NADPH) [EC:1.14.13.7] | Toluene degradation; Aminobenzoate degradation |
| K03381 | catA; catechol 1,2-dioxygenase [EC:1.13.11.1] | Benzoate degradation; Toluene degradation; Fluorobenzoate degradation |
| K03464 | catC; muconolactone D-isomerase [EC:5.3.3.4] | Benzoate degradation |
| K03862 | vanA; vanillate monooxygenase [EC:1.14.13.82] | Aminobenzoate degradation |
| K03863 | vanB; vanillate monooxygenase ferredoxin subunit | Aminobenzoate degradation |
| K04072 | adhE; acetaldehyde dehydrogenase / alcohol dehydrogenase [EC:1.2.1.10 1.1.1.1] | Naphthalene degradation |
| K04073 | mhpF; acetaldehyde dehydrogenase [EC:1.2.1.10] | Benzoate degradation; Xylene degradation |
| K04098 | chqB; hydroxyquinol 1,2-dioxygenase [EC:1.13.11.37] | Benzoate degradation |
| K04099 | desB, galA; gallate dioxygenase [EC:1.13.11.57] | Aminobenzoate degradation |
| K04100 | ligA; protocatechuate 4,5-dioxygenase, alpha chain [EC:1.13.11.8] | Benzoate degradation; Polycyclic aromatic hydrocarbon degradation; Aminobenzoate degradation |
| K04101 | ligB; protocatechuate 4,5-dioxygenase, beta chain [EC:1.13.11.8] | Benzoate degradation; Polycyclic aromatic hydrocarbon degradation; Aminobenzoate degradation |
| K04102 | pht5; 4,5-dihydroxyphthalate decarboxylase [EC:4.1.1.55] | Polycyclic aromatic hydrocarbon degradation |
| K04105 | hbaA; 4-hydroxybenzoate-CoA ligase [EC:6.2.1.27 6.2.1.25] | Benzoate degradation; Aminobenzoate degradation |
| K04107 | hcrC, hbaB; 4-hydroxybenzoyl-CoA reductase subunit gamma [EC:1.1.7.1] | Benzoate degradation; Aminobenzoate degradation |
| K04108 | hcrA, hbaC; 4-hydroxybenzoyl-CoA reductase subunit alpha [EC:1.1.7.1] | Benzoate degradation; Aminobenzoate degradation |
| K04109 | hcrB, hbaD; 4-hydroxybenzoyl-CoA reductase subunit beta [EC:1.1.7.1] | Benzoate degradation; Aminobenzoate degradation |
| K04110 | badA; benzoate-CoA ligase [EC:6.2.1.25] | Benzoate degradation; Aminobenzoate degradation |
| K04112 | bcrC, badD; benzoyl-CoA reductase subunit C [EC:1.3.7.8] | Benzoate degradation |
| K04113 | bcrB, badE; benzoyl-CoA reductase subunit B [EC:1.3.7.8] | Benzoate degradation |
| K04114 | bcrA, badF; benzoyl-CoA reductase subunit A [EC:1.3.7.8] | Benzoate degradation |
| K04115 | bcrD, badG; benzoyl-CoA reductase subunit D [EC:1.3.7.8] | Benzoate degradation |
| K04116 | aliA; cyclohexanecarboxylate-CoA ligase [EC:6.2.1.-] | Benzoate degradation |
| K04117 | aliB; cyclohexanecarboxyl-CoA dehydrogenase [EC:1.3.99.-] | Benzoate degradation |
| K04755 | fdx; ferredoxin, 2Fe-2S | Benzoate degradation |
| K05549 | benA-xylX; benzoate/toluate 1,2-dioxygenase subunit alpha [EC:1.14.12.10 1.14.12.-] | Benzoate degradation; Xylene degradation; Fluorobenzoate degradation |
| K05550 | benB-xylY; benzoate/toluate 1,2-dioxygenase subunit beta [EC:1.14.12.10 1.14.12.-] | Benzoate degradation; Xylene degradation; Fluorobenzoate degradation |
| K05783 | benD-xylL; dihydroxycyclohexadiene carboxylate dehydrogenase [EC:1.3.1.25 1.3.1.-] | Benzoate degradation; Xylene degradation; Fluorobenzoate degradation |
| K05784 | benC-xylZ; benzoate/toluate 1,2-dioxygenase reductase component [EC:1.18.1.-] | Benzoate degradation; Xylene degradation; Fluorobenzoate degradation |
| K05797 | pchF; 4-cresol dehydrogenase (hydroxylating) flavoprotein subunit [EC:1.17.9.1] | Toluene degradation |
| K06281 | hyaB, hybC; hydrogenase large subunit [EC:1.12.99.6] | Nitrotoluene degradation |
| K06282 | hyaA, hybO; hydrogenase small subunit [EC:1.12.99.6] | Nitrotoluene degradation |
| K07104 | catE; catechol 2,3-dioxygenase [EC:1.13.11.2] | Benzoate degradation; Xylene degradation; Styrene degradation |
| K07516 | fadN; 3-hydroxyacyl-CoA dehydrogenase [EC:1.1.1.35] | Benzoate degradation |
| K07534 | badK; cyclohex-1-ene-1-carboxyl-CoA hydratase [EC:4.2.1.-] | Benzoate degradation |
| K07535 | badH; 2-hydroxycyclohexanecarboxyl-CoA dehydrogenase [EC:1.1.1.-] | Benzoate degradation |
| K07536 | badI; 2-ketocyclohexanecarboxyl-CoA hydrolase [EC:3.1.2.-] | Benzoate degradation |
| K07537 | dch; cyclohexa-1,5-dienecarbonyl-CoA hydratase [EC:4.2.1.100] | Benzoate degradation |
| K07538 | had; 6-hydroxycyclohex-1-ene-1-carbonyl-CoA dehydrogenase [EC:1.1.1.368] | Benzoate degradation |
| K07539 | oah; 6-oxocyclohex-1-ene-carbonyl-CoA hydrolase [EC:3.7.1.21] | Benzoate degradation |
| K07543 | bbsE; benzylsuccinate CoA-transferase BbsE subunit [EC:2.8.3.15] | Toluene degradation |
| K07544 | bbsF; benzylsuccinate CoA-transferase BbsF subunit [EC:2.8.3.15] | Toluene degradation |
| K07545 | bbsG; (R)-benzylsuccinyl-CoA dehydrogenase [EC:1.3.8.3] | Toluene degradation |
| K07546 | bbsH; (E)-benzylidenesuccinyl-CoA hydratase [EC:4.2.1.180] | Toluene degradation |
| K07547 | bbsC; (2S)-[(R)-hydroxy(phenyl)methyl]-succinyl-CoA dehydrogenase BbsC subunit [EC:1.1.1.429] | Toluene degradation |
| K07549 | bbsA; benzoylsuccinyl-CoA thiolase BbsA subunit [EC:2.3.1.310] | Toluene degradation |
| K07550 | bbsB; benzoylsuccinyl-CoA thiolase BbsB subunit [EC:2.3.1.310] | Toluene degradation |
| K07823 | pcaF; 3-oxoadipyl-CoA thiolase [EC:2.3.1.174] | Benzoate degradation |
| K08295 | abmG; 2-aminobenzoate-CoA ligase [EC:6.2.1.32] | Aminobenzoate degradation |
| K09461 | E1.14.13.40; anthraniloyl-CoA monooxygenase [EC:1.14.13.40] | Aminobenzoate degradation |
| K10215 | ethA; monooxygenase [EC:1.14.13.-] | Aminobenzoate degradation |
| K10216 | dmpD, xylF; 2-hydroxymuconate-semialdehyde hydrolase [EC:3.7.1.9] | Benzoate degradation; Xylene degradation; Styrene degradation |
| K10217 | dmpC, xylG, praB; aminomuconate-semialdehyde/2-hydroxymuconate-6-semialdehyde dehydrogenase [EC:1.2.1.32 1.2.1.85] | Benzoate degradation; Xylene degradation |
| K10218 | ligK, galC; 4-hydroxy-4-methyl-2-oxoglutarate aldolase [EC:4.1.3.17] | Benzoate degradation |
| K10219 | ligC; 2-hydroxy-4-carboxymuconate semialdehyde hemiacetal dehydrogenase [EC:1.1.1.312] | Benzoate degradation; Aminobenzoate degradation |
| K10220 | ligJ; 4-oxalmesaconate hydratase [EC:4.2.1.83] | Benzoate degradation |
| K10221 | ligI; 2-pyrone-4,6-dicarboxylate lactonase [EC:3.1.1.57] | Benzoate degradation; Aminobenzoate degradation |
| K10616 | cymAa; p-cymene methyl-monooxygenase [EC:1.14.15.25] | Xylene degradation |
| K10619 | cmtAb; p-cumate 2,3-dioxygenase subunit alpha [EC:1.14.12.25] | Xylene degradation |
| K10620 | cmtB; 2,3-dihydroxy-2,3-dihydro-p-cumate dehydrogenase [EC:1.3.1.58] | Xylene degradation |
| K10621 | cmtC, dhbA; 2,3-dihydroxy-p-cumate/2,3-dihydroxybenzoate 3,4-dioxygenase [EC:1.13.11.- 1.13.11.14] | Benzoate degradation; Xylene degradation |
| K10622 | cmtD, dhbB; HCOMODA/2-hydroxy-3-carboxy-muconic semialdehyde decarboxylase [EC:4.1.1.-] | Benzoate degradation; Xylene degradation |
| K10623 | cmtE; HOMODA hydrolase [EC:3.7.1.-] | Xylene degradation |
| K10679 | nfnB, nfsB; nitroreductase / dihydropteridine reductase [EC:1.-.-.- 1.5.1.34] | Nitrotoluene degradation |
| K10680 | nemA; N-ethylmaleimide reductase [EC:1.-.-.-] | Nitrotoluene degradation |
| K11180 | dsrA; dissimilatory sulfite reductase alpha subunit [EC:1.8.1.22] | Nitrotoluene degradation |
| K11181 | dsrB; dissimilatory sulfite reductase beta subunit [EC:1.8.1.22] | Nitrotoluene degradation |
| K11945 | phdF; extradiol dioxygenase [EC:1.13.11.-] | Polycyclic aromatic hydrocarbon degradation |
| K11949 | phdJ; 4-(2-carboxyphenyl)-2-oxobut-3-enoate aldolase [EC:4.1.2.34] | Polycyclic aromatic hydrocarbon degradation |
| K12508 | fcs; trans-feruloyl-CoA synthase [EC:6.2.1.34] | Aminobenzoate degradation |
| K13953 | adhP; alcohol dehydrogenase, propanol-preferring [EC:1.1.1.1] | Naphthalene degradation |
| K13954 | yiaY; alcohol dehydrogenase [EC:1.1.1.1] | Naphthalene degradation |
| K14333 | DHBD; 2,3-dihydroxybenzoate decarboxylase [EC:4.1.1.46] | Benzoate degradation; Aminobenzoate degradation |
| K14338 | cypD_E, CYP102A, CYP505; cytochrome P450 / NADPH-cytochrome P450 reductase [EC:1.14.14.1 1.6.2.4] | Aminobenzoate degradation |
| K14579 | nahAc, ndoB, nbzAc, dntAc; naphthalene 1,2-dioxygenase subunit alpha [EC:1.14.12.12 1.14.12.23 1.14.12.24] | Ethylbenzene degradation; Naphthalene degradation; Polycyclic aromatic hydrocarbon degradation; Aminobenzoate degradation; Nitrotoluene degradation |
| K14581 | nahAa, nagAa, ndoR, nbzAa, dntAa; naphthalene 1,2-dioxygenase ferredoxin reductase component [EC:1.18.1.7] | Ethylbenzene degradation; Naphthalene degradation; Polycyclic aromatic hydrocarbon degradation; Aminobenzoate degradation; Nitrotoluene degradation |
| K14582 | nahB, doxE; cis-1,2-dihydro-1,2-dihydroxynaphthalene/dibenzothiophene dihydrodiol dehydrogenase [EC:1.3.1.29 1.3.1.60] | Naphthalene degradation; Polycyclic aromatic hydrocarbon degradation |
| K14584 | nahD; 2-hydroxychromene-2-carboxylate isomerase [EC:5.99.1.4] | Naphthalene degradation |
| K14585 | nahE; trans-o-hydroxybenzylidenepyruvate hydratase-aldolase [EC:4.1.2.45] | Naphthalene degradation |
| K14748 | etbAa; ethylbenzene dioxygenase subunit alpha [EC:1.14.12.-] | Ethylbenzene degradation |
| K14749 | etbAb; ethylbenzene dioxygenase subunit beta [EC:1.14.12.-] | Ethylbenzene degradation |
| K14750 | etbAc; ethylbenzene dioxygenase ferredoxin component | Ethylbenzene degradation |
| K14751 | etbC; 2,3-dihydroxyethylbenzene 1,2-dioxygenase [EC:1.13.11.-] | Ethylbenzene degradation |
| K15054 | mdlB; (S)-mandelate dehydrogenase [EC:1.1.99.31] | Aminobenzoate degradation |
| K15056 | nbaB; 2-hydroxylaminobenzoate mutase [EC:5.4.99.-] | Aminobenzoate degradation |
| K15060 | ligXa; 5,5'-dehydrodivanillate O-demethylase oxygenase subunit [EC:1.14.13.-] | Aminobenzoate degradation |
| K15063 | ligW; 5-carboxyvanillate decarboxylase | Aminobenzoate degradation |
| K15066 | ligM; vanillate/3-O-methylgallate O-demethylase [EC:2.1.1.341] | Aminobenzoate degradation |
| K15511 | boxA; benzoyl-CoA 2,3-epoxidase subunit A [EC:1.14.13.208] | Benzoate degradation |
| K15512 | boxB; benzoyl-CoA 2,3-epoxidase subunit B [EC:1.14.13.208] | Benzoate degradation |
| K15513 | boxC; benzoyl-CoA-dihydrodiol lyase [EC:4.1.2.44] | Benzoate degradation |
| K15514 | boxD; 3,4-dehydroadipyl-CoA semialdehyde dehydrogenase [EC:1.2.1.77] | Benzoate degradation |
| K15757 | xylM; toluene methyl-monooxygenase [EC:1.14.15.26] | Xylene degradation; Toluene degradation |
| K15758 | xylA; toluene methyl-monooxygenase electron transfer component [EC:1.18.1.3] | Xylene degradation; Toluene degradation |
| K15760 | tmoA, tbuA1, touA; toluene monooxygenase system protein A [EC:1.14.13.236 1.14.13.-] | Toluene degradation |
| K15761 | tmoB, tbuU, touB; toluene monooxygenase system protein B [EC:1.14.13.236 1.14.13.-] | Toluene degradation |
| K15762 | tmoC, tbuB, touC; toluene monooxygenase system ferredoxin subunit | Toluene degradation |
| K15763 | tmoD, tbuV, touD; toluene monooxygenase system protein D [EC:1.14.13.236 1.14.13.-] | Toluene degradation |
| K15764 | tmoE, tbuA2, touE; toluene monooxygenase system protein E [EC:1.14.13.236 1.14.13.-] | Toluene degradation |
| K15765 | tmoF, tbuC, touF; toluene monooxygenase electron transfer component [EC:1.18.1.3] | Toluene degradation |
| K16171 | faaH; fumarylacetoacetate (FAA) hydrolase [EC:3.7.1.2] | Styrene degradation |
| K16173 | acd; glutaryl-CoA dehydrogenase (non-decarboxylating) [EC:1.3.99.32] | Benzoate degradation |
| K16239 | bsdC; 4-hydroxybenzoate decarboxylase subunit C [EC:4.1.1.61] | Aminobenzoate degradation |
| K16242 | dmpN, poxD, tomA3; phenol/toluene 2-monooxygenase (NADH) P3/A3 [EC:1.14.13.244 1.14.13.243] | Benzoate degradation; Toluene degradation |
| K16243 | dmpL, poxB, tomA1; phenol/toluene 2-monooxygenase (NADH) P1/A1 [EC:1.14.13.244 1.14.13.243] | Benzoate degradation; Toluene degradation |
| K16244 | dmpM, poxC, tomA2; phenol/toluene 2-monooxygenase (NADH) P2/A2 [EC:1.14.13.244 1.14.13.243] | Benzoate degradation; Toluene degradation |
| K16245 | dmpO, poxE, tomA4; phenol/toluene 2-monooxygenase (NADH) P4/A4 [EC:1.14.13.244 1.14.13.243] | Benzoate degradation; Toluene degradation |
| K16246 | dmpP, poxF, tomA5; phenol/toluene 2-monooxygenase (NADH) P5/A5 [EC:1.14.13.244 1.14.13.243] | Benzoate degradation; Toluene degradation |
| K16249 | dmpK, poxA, tomA0; phenol/toluene 2-monooxygenase (NADH) P0/A0 | Benzoate degradation; Toluene degradation |
| K16303 | cmtAc; p-cumate 2,3-dioxygenase subunit beta [EC:1.14.12.25] | Xylene degradation |
| K16304 | cmtAd; p-cumate 2,3-dioxygenase ferredoxin component | Xylene degradation |
| K16319 | andAc; anthranilate 1,2-dioxygenase large subunit [EC:1.14.12.1] | Aminobenzoate degradation |
| K16320 | andAd; anthranilate 1,2-dioxygenase small subunit [EC:1.14.12.1] | Aminobenzoate degradation |
| K16514 | galD; 4-oxalomesaconate tautomerase [EC:5.3.2.8] | Benzoate degradation |
| K16515 | galB; 4-oxalomesaconate hydratase [EC:4.2.1.83] | Benzoate degradation |
| K18067 | pht4; phthalate 4,5-cis-dihydrodiol dehydrogenase [EC:1.3.1.64] | Polycyclic aromatic hydrocarbon degradation |
| K18068 | pht3; phthalate 4,5-dioxygenase [EC:1.14.12.7] | Polycyclic aromatic hydrocarbon degradation |
| K18069 | pht2; phthalate 4,5-dioxygenase reductase component [EC:1.18.1.-] | Polycyclic aromatic hydrocarbon degradation |
| K18076 | tphB; 1,2-dihydroxy-3,5-cyclohexadiene-1,4-dicarboxylate dehydrogenase [EC:1.3.1.53] | Polycyclic aromatic hydrocarbon degradation |
| K18077 | tphA1; terephthalate 1,2-dioxygenase reductase component [EC:1.18.1.-] | Polycyclic aromatic hydrocarbon degradation |
| K18092 | etbD; 2-hydroxy-6-oxo-octa-2,4-dienoate hydrolase [EC:3.7.1.-] | Ethylbenzene degradation |
| K18242 | nagG; salicylate 5-hydroxylase large subunit [EC:1.14.13.172] | Naphthalene degradation |
| K18243 | nagH; salicylate 5-hydroxylase small subunit [EC:1.14.13.172] | Naphthalene degradation |
| K18252 | phtAb; phthalate 3,4-dioxygenase subunit beta [EC:1.14.12.-] | Polycyclic aromatic hydrocarbon degradation |
| K18256 | phtC; 3,4-dihydroxyphthalate decarboxylase [EC:4.1.1.69] | Polycyclic aromatic hydrocarbon degradation |
| K18275 | phdK; 2-formylbenzoate dehydrogenase [EC:1.2.1.78] | Polycyclic aromatic hydrocarbon degradation |
| K18293 | cymAb; p-cymene methyl-monooxygenase electron transfer component [EC:1.18.1.3] | Xylene degradation |
| K18364 | bphH, xylJ, tesE; 2-oxopent-4-enoate/cis-2-oxohex-4-enoate hydratase [EC:4.2.1.80 4.2.1.132] | Benzoate degradation; Xylene degradation |
| K18366 | bphJ, xylQ, nahO, tesF; acetaldehyde/propanal dehydrogenase [EC:1.2.1.10 1.2.1.87] | Benzoate degradation; Xylene degradation |
| K18383 | ferB; feruloyl-CoA hydratase/lyase [EC:4.1.2.61] | Aminobenzoate degradation |
| K19516 | bamC; benzoyl-CoA reductase subunit BamC [EC:1.3.-.-] | Benzoate degradation |
| K20199 | pchA; 4-hydroxybenzaldehyde dehydrogenase (NADP+) [EC:1.2.1.96] | Toluene degradation |
| K20200 | pchC; 4-cresol dehydrogenase (hydroxylating) cytochrome subunit | Toluene degradation |
| K20218 | hipH; 4-hydroxyisophthalate hydroxylase | Toluene degradation |
| K20458 | hcl; 3-hydroxybenzoate/4-hydroxybenzoate---CoA ligase [EC:6.2.1.37 6.2.1.27] | Benzoate degradation; Aminobenzoate degradation |
| K20509 | madB, oadB, gcdB, mmdB; carboxybiotin decarboxylase [EC:7.2.4.1] | Benzoate degradation |
| K20807 | nthB; nitrile hydratase subunit beta [EC:4.2.1.84] | Aminobenzoate degradation; Styrene degradation; Fluorobenzoate degradation |
| K20941 | graF; gamma-resorcylate decarboxylase [EC:4.1.1.103] | Benzoate degradation |
| K21725 | pnpA; 4-nitrocatechol/4-nitrophenol 4-monooxygenase [EC:1.14.13.166 1.14.13.167] | Aminobenzoate degradation |
| K21759 | bsdD; vanillate/4-hydroxybenzoate decarboxylase subunit D [EC:4.1.1.- 4.1.1.61] | Aminobenzoate degradation |
| K22270 | nagX; 3-hydroxybenzoate 6-monooxygenase [EC:1.14.13.24] | Benzoate degradation |
| K22553 | CYP199A2; 4-methoxybenzoate monooxygenase (O-demethylating) [EC:1.14.99.15] | Benzoate degradation |
| K22958 | lpdC; gallate decarboxylase subunit C [EC:4.1.1.59] | Aminobenzoate degradation |
| K22960 | lpdD; gallate decarboxylase subunit D | Aminobenzoate degradation |
| K23351 | gcdC, mmdC; glutaconyl-CoA/methylmalonyl-CoA decarboxylase subunit gamma | Benzoate degradation |
| K23352 | gcdD, mmdD; glutaconyl-CoA/methylmalonyl-CoA decarboxylase subunit delta | Benzoate degradation |
| K23548 | hupU; uptake hydrogenase small subunit [EC:1.12.99.6] | Nitrotoluene degradation |
| K23549 | hupV; uptake hydrogenase large subunit [EC:1.12.99.6] | Nitrotoluene degradation |
| K25916 | mtvB, mtoB; methoxylated aromatic compound---corrinoid protein Co-methyltransferase [EC:2.1.1.382] | Aminobenzoate degradation |
| K25932 | ppcA; phenyl-phosphate phosphatase/carboxylase subunit alpha [EC:4.1.1.123] | Aminobenzoate degradation |
| K25933 | ppcB; phenyl-phosphate phosphatase/carboxylase subunit beta [EC:4.1.1.123] | Aminobenzoate degradation |
| K25934 | ppcC; phenyl-phosphate phosphatase/carboxylase subunit gamma [EC:4.1.1.123] | Aminobenzoate degradation |
| K25936 | ppsA; phenol phosphorylase subunit alpha [EC:2.7.1.238] | Aminobenzoate degradation |
| K25937 | ppsB; phenol phosphorylase subunit beta [EC:2.7.1.238] | Aminobenzoate degradation |
| K25938 | ppsC; phenol phosphorylase subunit delta gamma [EC:2.7.1.238] | Aminobenzoate degradation |
| K27196 | dsrL; dissimilatory sulfite reductase flavoprotein subunit | Nitrotoluene degradation |
| K27848 | E2.3.1.318; 3-oxoadipate:acetyl-CoA acetyltransferase [EC:2.3.1.318] | Benzoate degradation |

**Table S6. Well-level composite indices and operational attenuation-state classifications.**

| **Well** | **Pollution pressure** | **Biological response** | **NAPI** | **Pressure-response mismatch** | **Taxonomic-functional diversity** | **Functional completeness** | **Operational attenuation state** |
| --- | --- | --- | --- | --- | --- | --- | --- |
| W1 | 1.132 | 0.520 | 0.749 | -0.345 | 0.365 | 0.457 | Active attenuation under high pressure |
| W2 | 1.499 | 0.495 | 0.768 | -0.816 | 0.403 | 0.363 | Active attenuation under high pressure |
| W3 | 0.711 | 0.855 | 0.812 | 0.766 | 0.424 | 0.553 | Active attenuation under high pressure |
| W4 | 0.435 | 0.162 | 0.609 | -0.204 | 0.349 | 0.404 | Active attenuation under high pressure |
| W5 | 0.368 | 0.121 | 0.587 | -0.202 | 0.411 | 0.271 | Transition attenuation zone |
| W6 | 0.356 | 0.184 | 0.605 | -0.071 | 0.370 | 0.262 | Transition attenuation zone |
| W7 | 1.042 | 0.983 | 0.857 | 0.621 | 0.328 | 0.271 | Active attenuation under high pressure |
| W8 | -0.660 | -0.453 | 0.364 | -0.078 | 0.217 | 0.171 | Low-impact/background |
| W9 | -0.287 | -0.750 | 0.298 | -1.062 | 0.523 | 0.386 | Transition attenuation zone |
| W10 | -0.087 | -0.186 | 0.477 | -0.245 | 0.274 | 0.259 | Transition attenuation zone |
| W11 | -1.113 | 0.089 | 0.476 | 1.457 | 0.380 | 0.322 | Residual/recovery with legacy response |
| W12 | -0.609 | -0.876 | 0.243 | -0.924 | 0.517 | 0.394 | Low-impact/background |
| W13 | -1.574 | -0.346 | 0.319 | 1.182 | 0.920 | 0.963 | Residual/recovery with legacy response |
| W14 | -0.940 | -0.287 | 0.381 | 0.557 | 0.515 | 0.394 | Residual/recovery with legacy response |
| W15 | -0.273 | -0.511 | 0.360 | -0.635 | 0.766 | 0.886 | Transition attenuation zone |

**Table S7. Constituents of the functional modules used in the study.**

| **Functional module** | **Genera included** | **KEGG pathways included** |
| --- | --- | --- |
| Aromatic hydrocarbon response module | Acidovorax; Pseudoxanthomonas; Aromatoleum; Pseudomonas; Thauera; Comamonas; Diaphorobacter | Benzoate degradation;Xylene degradation;Toluene degradation;Ethylbenzene degradation;Styrene degradation |
| Plume-margin redox transition module | Hydrogenophaga; Azospira;Polaromonas;Variovorax;Comamonas;Sulfuritalea | Nitrogen metabolism;Sulfur metabolism |
| Anaerobic terminal-process module | Methanothrix; Sulfurospirillum;Dehalobacter;Desulfovibrio;Azoarcus | Methane metabolism;Sulfur metabolism;Benzoate degradation |
| PAH/naphthalene residual module | Sphingomonas;Novosphingobium;Xanthobacter;Polaromonas | Naphthalene degradation;Polycyclic aromatic hydrocarbon degradation;Fluorobenzoate degradation |

**Table S8. Spearman correlations between measured geochemical constituents and metagenome-derived hydrocarbon-degradation potential across the 15 groundwater wells.**

| Functional metric / degradation pathway | Geochemical variable | Spearman rho | Permutation P | BH-adjusted q | FDR significant |
| --- | --- | --- | --- | --- | --- |
| Aggregate degradation-pathway abundance | pH | .760 | .002 | .016 | Yes |
| Aggregate degradation-pathway abundance | ORP (mV) | .438 | .109 | .216 | No |
| Aggregate degradation-pathway abundance | Nitrate (ppm) | -.258 | .328 | .375 | No |
| Aggregate degradation-pathway abundance | TOC (ppm) | -.379 | .135 | .216 | No |
| Degradation-associated functional diversity | pH | .687 | .009 | .036 | Yes |
| Degradation-associated functional diversity | ORP (mV) | .391 | .165 | .220 | No |
| Degradation-associated functional diversity | Nitrate (ppm) | .042 | .883 | .883 | No |
| Degradation-associated functional diversity | TOC (ppm) | -.464 | .097 | .216 | No |
| Benzoate degradation | pH | .705 | .008 | .080 | No |
| Benzoate degradation | ORP (mV) | .522 | .060 | .225 | No |
| Benzoate degradation | Nitrate (ppm) | -.197 | .464 | .714 | No |
| Benzoate degradation | TOC (ppm) | -.100 | .705 | .855 | No |
| Xylene degradation | pH | .512 | .062 | .225 | No |
| Xylene degradation | ORP (mV) | .189 | .502 | .744 | No |
| Xylene degradation | Nitrate (ppm) | .002 | .998 | .998 | No |
| Xylene degradation | TOC (ppm) | .011 | .968 | .998 | No |
| Toluene degradation | pH | .433 | .111 | .317 | No |
| Toluene degradation | ORP (mV) | .145 | .597 | .815 | No |
| Toluene degradation | Nitrate (ppm) | .013 | .974 | .998 | No |
| Toluene degradation | TOC (ppm) | -.071 | .809 | .899 | No |
| Ethylbenzene degradation | pH | .821 | .001 | .020 | Yes |
| Ethylbenzene degradation | ORP (mV) | .538 | .042 | .187 | No |
| Ethylbenzene degradation | Nitrate (ppm) | -.329 | .237 | .474 | No |
| Ethylbenzene degradation | TOC (ppm) | -.343 | .226 | .474 | No |
| Naphthalene degradation | pH | .778 | .003 | .040 | Yes |
| Naphthalene degradation | ORP (mV) | .608 | .018 | .144 | No |
| Naphthalene degradation | Nitrate (ppm) | -.140 | .611 | .815 | No |
| Naphthalene degradation | TOC (ppm) | -.154 | .592 | .815 | No |
| Polycyclic aromatic hydrocarbon degradation | pH | .224 | .400 | .667 | No |
| Polycyclic aromatic hydrocarbon degradation | ORP (mV) | .375 | .158 | .421 | No |
| Polycyclic aromatic hydrocarbon degradation | Nitrate (ppm) | -.129 | .648 | .836 | No |
| Polycyclic aromatic hydrocarbon degradation | TOC (ppm) | -.229 | .446 | .714 | No |
| Aminobenzoate degradation | pH | .052 | .852 | .921 | No |
| Aminobenzoate degradation | ORP (mV) | .073 | .798 | .899 | No |
| Aminobenzoate degradation | Nitrate (ppm) | -.468 | .073 | .240 | No |
| Aminobenzoate degradation | TOC (ppm) | -.496 | .078 | .240 | No |
| Nitrotoluene degradation | pH | -.302 | .302 | .549 | No |
| Nitrotoluene degradation | ORP (mV) | -.529 | .038 | .187 | No |
| Nitrotoluene degradation | Nitrate (ppm) | -.299 | .265 | .505 | No |
| Nitrotoluene degradation | TOC (ppm) | -.361 | .194 | .468 | No |
| Styrene degradation | pH | .336 | .199 | .468 | No |
| Styrene degradation | ORP (mV) | -.118 | .680 | .850 | No |
| Styrene degradation | Nitrate (ppm) | -.071 | .784 | .899 | No |
| Styrene degradation | TOC (ppm) | -.586 | .025 | .167 | No |
| Fluorobenzoate degradation | pH | .810 | .001 | .020 | Yes |
| Fluorobenzoate degradation | ORP (mV) | .338 | .212 | .471 | No |
| Fluorobenzoate degradation | Nitrate (ppm) | -.257 | .343 | .597 | No |
| Fluorobenzoate degradation | TOC (ppm) | -.543 | .039 | .187 | No |

Note: Two-sided Spearman permutation tests used 999 permutations. Benjamini-Hochberg (BH) correction was applied across the eight primary tests and across all 40 pathway-by-geochemistry tests. Aggregate degradation-pathway abundance represents the summed relative abundance of the ten listed pathways. The degradation-associated functional diversity was derived from pathway- and KO-level richness and Shannon diversity.

**Table S9. Supplementary species-level multivariate tests.**

| **Level** | **Analysis** | **Distance matrix or grouping** | **Effect size** | **P** | **Pseudo-F** |
| --- | --- | --- | --- | --- | --- |
| Species | Mantel test | taxonomic-functional diversity | 0.415 | 0.006 |  |
| Species | Mantel test | spatial distance | 0.110 | 0.440 |  |
| Species | Mantel test | module | 0.648 | 0.001 |  |
| Species | PERMANOVA | attenuation state | 0.343 | 0.012 | 1.914 |

**Table S10. Stability of NAPI and taxonomic-functional diversity rankings across alternative weighting scenarios.**

| **Index** | **Well** | **Mean score** | **SD** | **Median rank** | **Best rank** | **Worst rank** | **Top-three frequency** | **Top-five frequency** |
| --- | --- | --- | --- | --- | --- | --- | --- | --- |
| NAPI | W7 | 0.857 | 0.027 | 1.000 | 1.000 | 4.000 | 0.960 | 1.000 |
|  | W3 | 0.812 | 0.026 | 2.000 | 1.000 | 4.000 | 0.984 | 1.000 |
|  | W2 | 0.769 | 0.059 | 3.000 | 1.000 | 5.000 | 0.688 | 1.000 |
|  | W1 | 0.749 | 0.037 | 4.000 | 2.000 | 4.000 | 0.356 | 1.000 |
|  | W4 | 0.609 | 0.048 | 6.000 | 5.000 | 8.000 | 0.000 | 0.415 |
|  | W6 | 0.605 | 0.033 | 6.000 | 5.000 | 8.000 | 0.000 | 0.285 |
|  | W5 | 0.586 | 0.081 | 7.000 | 3.000 | 14.000 | 0.012 | 0.289 |
|  | W10 | 0.477 | 0.047 | 8.000 | 8.000 | 12.000 | 0.000 | 0.000 |
|  | W11 | 0.476 | 0.047 | 9.000 | 5.000 | 11.000 | 0.000 | 0.004 |
|  | W8 | 0.366 | 0.066 | 11.000 | 7.000 | 14.000 | 0.000 | 0.000 |
|  | W14 | 0.381 | 0.031 | 11.000 | 10.000 | 13.000 | 0.000 | 0.000 |
|  | W15 | 0.361 | 0.050 | 12.000 | 7.000 | 13.000 | 0.000 | 0.000 |
|  | W9 | 0.297 | 0.048 | 13.000 | 9.000 | 15.000 | 0.000 | 0.000 |
|  | W13 | 0.320 | 0.089 | 13.000 | 5.000 | 15.000 | 0.000 | 0.008 |
|  | W12 | 0.244 | 0.047 | 15.000 | 13.000 | 15.000 | 0.000 | 0.000 |
| Taxonomic-functional diversity | W13 | 0.919 | 0.028 | 1.000 | 1.000 | 1.000 | 1.000 | 1.000 |
|  | W15 | 0.766 | 0.054 | 2.000 | 2.000 | 7.000 | 0.999 | 0.999 |
|  | W14 | 0.515 | 0.059 | 4.000 | 3.000 | 11.000 | 0.122 | 0.820 |
|  | W9 | 0.524 | 0.063 | 4.000 | 2.000 | 9.000 | 0.435 | 0.876 |
|  | W12 | 0.517 | 0.043 | 5.000 | 3.000 | 7.000 | 0.280 | 0.890 |
|  | W3 | 0.424 | 0.061 | 7.000 | 3.000 | 15.000 | 0.148 | 0.248 |
|  | W5 | 0.411 | 0.068 | 7.000 | 3.000 | 14.000 | 0.012 | 0.053 |
|  | W2 | 0.403 | 0.042 | 8.000 | 3.000 | 13.000 | 0.003 | 0.067 |
|  | W11 | 0.380 | 0.033 | 9.000 | 6.000 | 12.000 | 0.000 | 0.000 |
|  | W1 | 0.365 | 0.044 | 10.000 | 4.000 | 15.000 | 0.000 | 0.046 |
|  | W6 | 0.370 | 0.046 | 10.000 | 6.000 | 15.000 | 0.000 | 0.000 |
|  | W4 | 0.349 | 0.024 | 11.000 | 5.000 | 14.000 | 0.000 | 0.001 |
|  | W7 | 0.328 | 0.028 | 13.000 | 8.000 | 14.000 | 0.000 | 0.000 |
|  | W10 | 0.274 | 0.031 | 14.000 | 10.000 | 15.000 | 0.000 | 0.000 |
|  | W8 | 0.217 | 0.052 | 15.000 | 8.000 | 15.000 | 0.000 | 0.000 |

**Table S11. Exploratory genus-module associations. Nominal P values were obtained from two-sided permutation tests (999 permutations); q values were adjusted using the Benjamini-Hochberg procedure.**

| **Genus** | **Functional module** | **Spearman rho** | **Nominal P** | **FDR q** | **Reported degradation/attenuation relevance (reference)** |
| --- | --- | --- | --- | --- | --- |
| *Acidovorax* | Aromatic hydrocarbon response module | 0.746 | 0.002 | 0.080 | Phenanthrene/PAH degradation (Singleton et al., 2009) |
| *Acidovorax* | Anaerobic terminal-process module | -0.611 | 0.011 | 0.130 | Phenanthrene/PAH degradation (Singleton et al., 2009) |
| *Sphingopyxis* | Plume-margin redox transition module | 0.675 | 0.011 | 0.130 | Crude-oil, diesel, and kerosene degradation (Kim et al., 2014) |
| *Variovorax* | Plume-margin redox transition module | 0.625 | 0.013 | 0.130 | Aerobic benzene degradation in coal-tar-contaminated groundwater (Posman et al., 2017) |
| *Pseudoxanthomonas* | Aromatic hydrocarbon response module | 0.579 | 0.022 | 0.176 | Chrysene/PAH degradation (Nayak et al., 2011) |
| *Pseudoxanthomonas* | Plume-margin redox transition module | -0.593 | 0.028 | 0.187 | Chrysene/PAH degradation (Nayak et al., 2011) |
| *Hydrogenophaga* | Plume-margin redox transition module | 0.518 | 0.042 | 0.222 | Aerobic xylene degradation in a legacy BTEX-contaminated aquifer (Banerjee et al., 2022) |
| *Variovorax* | PAH/naphthalene residual module | 0.500 | 0.048 | 0.222 | Aerobic benzene degradation in coal-tar-contaminated groundwater (Posman et al., 2017) |
| *Pseudoxanthomonas* | Anaerobic terminal-process module | -0.500 | 0.050 | 0.222 | Chrysene/PAH degradation (Nayak et al., 2011) |
| *Sphingopyxis* | Aromatic hydrocarbon response module | -0.518 | 0.057 | 0.228 | Crude-oil, diesel, and kerosene degradation (Kim et al., 2014) |
| *Comamonas* | Plume-margin redox transition module | 0.486 | 0.065 | 0.236 | PAH degradation, including naphthalene and phenanthrene (Lu et al., 2022) |
| *Hydrogenophaga* | PAH/naphthalene residual module | 0.400 | 0.140 | 0.465 | Aerobic xylene degradation in a legacy BTEX-contaminated aquifer (Banerjee et al., 2022) |
| *Azospira* | PAH/naphthalene residual module | -0.404 | 0.151 | 0.465 | Association with anaerobic toluene-degrading consortia and nitrate-reducing contaminated-groundwater lineages (Jiang et al., 2023; Mikes et al., 2021) |
| *Comamonas* | PAH/naphthalene residual module | 0.354 | 0.173 | 0.494 | PAH degradation, including naphthalene and phenanthrene (Lu et al., 2022) |
| *Azospira* | Aromatic hydrocarbon response module | -0.346 | 0.191 | 0.509 | Association with anaerobic toluene-degrading consortia and nitrate-reducing contaminated-groundwater lineages (Jiang et al., 2023; Mikes et al., 2021) |
| *Sphingopyxis* | PAH/naphthalene residual module | 0.343 | 0.209 | 0.522 | Crude-oil, diesel, and kerosene degradation (Kim et al., 2014) |
| *Aromatoleum* | Anaerobic terminal-process module | 0.318 | 0.251 | 0.531 | Anaerobic aromatic-hydrocarbon degradation under denitrifying conditions in petroleum-plume systems (Bauer et al., 2009) |
| *Acidovorax* | Plume-margin redox transition module | -0.332 | 0.255 | 0.531 | Phenanthrene/PAH degradation (Singleton et al., 2009) |
| *Methanothrix* | Anaerobic terminal-process module | -0.311 | 0.270 | 0.531 | Methanogenic terminal processes in petroleum-hydrocarbon-contaminated aquifers (Kleikemper et al., 2005) |
| *Azospira* | Anaerobic terminal-process module | -0.304 | 0.279 | 0.531 | Association with anaerobic toluene-degrading consortia and nitrate-reducing contaminated-groundwater lineages (Jiang et al., 2023; Mikes et al., 2021) |
| *Polaromonas* | Aromatic hydrocarbon response module | 0.286 | 0.279 | 0.531 | Naphthalene degradation in contaminated sediment (Jeon et al., 2004) |
| *Methanothrix* | PAH/naphthalene residual module | -0.282 | 0.297 | 0.540 | Methanogenic terminal processes in petroleum-hydrocarbon-contaminated aquifers (Kleikemper et al., 2005) |
| *Aromatoleum* | Aromatic hydrocarbon response module | 0.268 | 0.350 | 0.597 | Anaerobic aromatic-hydrocarbon degradation under denitrifying conditions in petroleum-plume systems (Bauer et al., 2009) |
| *Pseudoxanthomonas* | PAH/naphthalene residual module | -0.232 | 0.358 | 0.597 | Chrysene/PAH degradation (Nayak et al., 2011) |
| *Sphingopyxis* | Anaerobic terminal-process module | 0.232 | 0.410 | 0.643 | Crude-oil, diesel, and kerosene degradation (Kim et al., 2014) |
| *Aromatoleum* | Plume-margin redox transition module | -0.221 | 0.418 | 0.643 | Anaerobic aromatic-hydrocarbon degradation under denitrifying conditions in petroleum-plume systems (Bauer et al., 2009) |
| *Comamonas* | Anaerobic terminal-process module | 0.221 | 0.436 | 0.646 | PAH degradation, including naphthalene and phenanthrene (Lu et al., 2022) |
| *Methanothrix* | Plume-margin redox transition module | -0.189 | 0.502 | 0.694 | Methanogenic terminal processes in petroleum-hydrocarbon-contaminated aquifers (Kleikemper et al., 2005) |
| *Comamonas* | Aromatic hydrocarbon response module | -0.189 | 0.503 | 0.694 | PAH degradation, including naphthalene and phenanthrene (Lu et al., 2022) |
| *Variovorax* | Anaerobic terminal-process module | 0.150 | 0.600 | 0.800 | Aerobic benzene degradation in coal-tar-contaminated groundwater (Posman et al., 2017) |
| *Polaromonas* | Plume-margin redox transition module | -0.143 | 0.631 | 0.814 | Naphthalene degradation in contaminated sediment (Jeon et al., 2004) |
| *Polaromonas* | Anaerobic terminal-process module | -0.104 | 0.713 | 0.842 | Naphthalene degradation in contaminated sediment (Jeon et al., 2004) |
| *Variovorax* | Aromatic hydrocarbon response module | -0.104 | 0.716 | 0.842 | Aerobic benzene degradation in coal-tar-contaminated groundwater (Posman et al., 2017) |
| *Hydrogenophaga* | Aromatic hydrocarbon response module | -0.096 | 0.727 | 0.842 | Aerobic xylene degradation in a legacy BTEX-contaminated aquifer (Banerjee et al., 2022) |
| *Hydrogenophaga* | Anaerobic terminal-process module | 0.104 | 0.737 | 0.842 | Aerobic xylene degradation in a legacy BTEX-contaminated aquifer (Banerjee et al., 2022) |
| *Polaromonas* | PAH/naphthalene residual module | 0.086 | 0.767 | 0.852 | Naphthalene degradation in contaminated sediment (Jeon et al., 2004) |
| *Aromatoleum* | PAH/naphthalene residual module | 0.036 | 0.897 | 0.946 | Anaerobic aromatic-hydrocarbon degradation under denitrifying conditions in petroleum-plume systems (Bauer et al., 2009) |
| *Azospira* | Plume-margin redox transition module | 0.032 | 0.909 | 0.946 | Association with anaerobic toluene-degrading consortia and nitrate-reducing contaminated-groundwater lineages (Jiang et al., 2023; Mikes et al., 2021) |
| *Acidovorax* | PAH/naphthalene residual module | -0.032 | 0.922 | 0.946 | Phenanthrene/PAH degradation (Singleton et al., 2009) |
| *Methanothrix* | Aromatic hydrocarbon response module | -0.018 | 0.951 | 0.951 | Methanogenic terminal processes in petroleum-hydrocarbon-contaminated aquifers (Kleikemper et al., 2005) |

**References cited in Table S9**

Singleton, D. R., Guzmán Ramirez, L., and Aitken, M. D. (2009). Characterization of a polycyclic aromatic hydrocarbon degradation gene cluster in a phenanthrene-degrading Acidovorax strain. Applied and Environmental Microbiology 75, 2613–2620. doi:10.1128/AEM.01955-08.

Kim, J., Kim, S. J., Kim, S. H., Kim, S. I., Moon, Y.-J., Park, S.-J., Kahng, H.-Y., and Chung, Y.-H. (2014). Draft genome sequence of Sphingopyxis sp. strain MWB1, a crude-oil-degrading marine bacterium. Genome Announcements 2(6), e01256-14. doi:10.1128/genomeA.01256-14.

Posman, K. M., DeRito, C. M., and Madsen, E. L. (2017). Benzene degradation by a Variovorax species within a coal tar-contaminated groundwater microbial community. Applied and Environmental Microbiology 83, e02658-16. doi:10.1128/AEM.02658-16.

Nayak, A. S., Sanganal, S. K., Mudde, S. K., Oblesha, A., and Karegoudar, T. B. (2011). A catabolic pathway for the degradation of chrysene by Pseudoxanthomonas sp. PNK-04. FEMS Microbiology Letters 320(2), 128–134. doi:10.1111/j.1574-6968.2011.02301.x.

Banerjee, S., Bedics, A., Harkai, P., Kriszt, B., Alpula, N., and Táncsics, A. (2022). Evaluating the aerobic xylene-degrading potential of the intrinsic microbial community of a legacy BTEX-contaminated aquifer by enrichment culturing coupled with multi-omics analysis: uncovering the role of Hydrogenophaga strains in xylene degradation. Environmental Science and Pollution Research 29, 28431–28445. doi:10.1007/s11356-021-18300-w.

Lu, Q., Sun, X., Jiang, Z., Cui, Y., Li, X., and Cui, J. (2022). Effects of Comamonas testosteroni on dissipation of polycyclic aromatic hydrocarbons and the response of endogenous bacteria for soil bioremediation. Environmental Science and Pollution Research 29(54), 82351–82364. doi:10.1007/s11356-022-21497-z.

Jiang, H., Chen, D., Zheng, D., and Xiao, Z. (2023). Anaerobic mineralization of toluene by enriched soil-free consortia with solid-phase humin as a terminal electron acceptor. Environmental Pollution 317, 120794. doi:10.1016/j.envpol.2022.120794.

Mikes, M. C., Martin, T. K., and Moe, W. M. (2021). Azospira inquinata sp. nov., a nitrate-reducing bacterium of the family Rhodocyclaceae isolated from contaminated groundwater. International Journal of Systematic and Evolutionary Microbiology 71(12), 005172. doi:10.1099/ijsem.0.005172.

Bauer, R. D., Rolle, M., Bauer, S., Eberhardt, C., Grathwohl, P., Kolditz, O., Meckenstock, R. U., and Griebler, C. (2009). Enhanced biodegradation by hydraulic heterogeneities in petroleum hydrocarbon plumes. Journal of Contaminant Hydrology 105(1–2), 56–68. doi:10.1016/j.jconhyd.2008.11.004.

Kleikemper, J., Pombo, S. A., Schroth, M. H., Sigler, W. V., Pesaro, M., and Zeyer, J. (2005). Activity and diversity of methanogens in a petroleum hydrocarbon-contaminated aquifer. Applied and Environmental Microbiology 71(1), 149–158. doi:10.1128/AEM.71.1.149-158.2005.

Jeon, C. O., Park, W., Ghiorse, W. C., and Madsen, E. L. (2004). Polaromonas naphthalenivorans sp. nov., a naphthalene-degrading bacterium from naphthalene-contaminated sediment. International Journal of Systematic and Evolutionary Microbiology 54(1), 93–97. doi:10.1099/ijs.0.02636-0.
